# Fatigue, quality of life and physical fitness following an exercise intervention in multiple myeloma survivors (MASCOT): an exploratory randomised Phase 2 trial utilising a modified Zelen design

**DOI:** 10.1038/s41416-020-0866-y

**Published:** 2020-05-21

**Authors:** Dimitrios A. Koutoukidis, Joanne Land, Allan Hackshaw, Malgorzata Heinrich, Orla McCourt, Rebecca J. Beeken, Stephanie Philpott, Dunnya DeSilva, Ali Rismani, Neil Rabin, Rakesh Popat, Charalampia Kyriakou, Xenofon Papanikolaou, Atul Mehta, Bruce Paton, Abigail Fisher, Kwee L. Yong

**Affiliations:** 1 Department of Behavioural Science and Health, University College London, London, UK; 2 Nuffield Department of Primary Care Health Sciences, University of Oxford, Oxford, UK; 3 NIHR Biomedical Research Centre, Oxford University Hospitals NHS Foundation Trust, Oxford, UK; 4 Cancer Research UK & UCL Cancer Trials Centre, University College London, London, UK; 5 Cancer Institute, University College London, London, UK; 6 Leeds Institute of Health Sciences, University of Leeds, Leeds, UK; 7 Department of Haematology, University College London Hospitals NHS Foundation Trust, London, UK; 8 Department of Haematology, Lister Hospital, Stevenage, UK; 9 Institute of Sport, Exercise and Health, London, UK

**Keywords:** Quality of life, Outcomes research, Rehabilitation, Myeloma

## Abstract

**Background:**

Exercise may improve fatigue in multiple myeloma survivors, but trial evidence is limited, and exercise may be perceived as risky in this older patient group with osteolytic bone destruction.

**Methods:**

In this Phase 2 Zelen trial, multiple myeloma survivors who had completed treatment at least 6 weeks ago, or were on maintenance only, were enrolled in a cohort study and randomly assigned to usual care or a 6-month exercise programme of tailored aerobic and resistance training. Outcome assessors and usual care participants were masked. The primary outcome was the FACIT-F fatigue score with higher scores denoting less fatigue.

**Results:**

During 2014–2016, 131 participants were randomised 3:1 to intervention (*n* = 89) or usual care (*n* = 42) to allow for patients declining allocation to the exercise arm. There was no difference between groups in fatigue at 3 months (between-group mean difference: 1.6 [95% CI: −1.1–4.3]) or 6 months (0.3 [95% CI: −2.6–3.1]). Muscle strength improved at 3 months (8.4 kg [95% CI: 0.5–16.3]) and 6 months (10.8 kg [95% CI: 1.2–20.5]). Using per-protocol analysis, cardiovascular fitness improved at 3 months (+1.2 ml/kg/min [95% CI: 0.3–3.7]). In participants with clinical fatigue (*n* = 17), there was a trend towards less fatigue with exercise over 6 months (6.3 [95% CI: −0.6–13.3]). There were no serious adverse events.

**Conclusions:**

Exercise appeared safe and improved muscle strength and cardiovascular fitness, but benefits in fatigue appeared limited to participants with clinical fatigue at baseline. Future studies should focus on patients with clinical fatigue.

**Clinical trial registration:**

The study was registered with ISRCTN (38480455) and is completed.

## Background

The survival of patients with multiple myeloma (MM) continues to improve with the use of increasingly effective multidrug regimens. However, the disease remains incurable, and patients continue to experience a high symptom burden, particularly fatigue, throughout the disease trajectory and even during treatment-free periods.^1^ Up to 90% of patients suffer from osteolytic bone disease, causing pain, fractures, vertebral collapse and spinal cord compression.^2^ Even when the disease has responded to chemotherapy, and patients enjoy a treatment-free interval, the sequelae of bone destruction (reduced physical functioning, loss of muscle mass and chronic pain) affect their quality of life (QoL).^2^ Fatigue is associated with greater impairment of daily activities and lower quality of life, as well as shorter progression-free and overall survival.^3,4^ Therefore, managing these symptoms may help this growing population improve their QoL.

One way of managing cancer-related fatigue is through exercise interventions, and a considerable evidence base exists to support guidelines recommending exercise for survivors with fatigue.^5^ However, most of the evidence comes from patients with solid tumours, primarily breast cancer. A recent Cochrane review of 18 randomised clinical trials (RCTs) in survivors of haematological cancers found that most studies were poorly reported, generally of low quality, affected by bias due to contamination as blinding is not possible, and had insufficient follow-up.^6^

Exercise interventions specifically in MM survivors are sparse. Data suggest that physical activity is positively associated with QoL, but only about a fifth of MM survivors are meeting the exercise guidelines.^7^ Although MM patients express a desire for exercise support, they also fear injury and pain,^8^ which is understandable given the risk of fracture. Only two RCTs have focused exclusively on MM patients, and both evaluated home-based exercise programmes while undergoing first-line anticancer therapy.^9,10^ One RCT in 24 patients reported improved muscle strength and sleep.^9^ The other trial in 187 patients found no benefits for fatigue, sleep and aerobic capacity,^10^ potentially due to the usual care participants receiving exercise instructions from their clinician. A major problem with traditional behavioural change trials is that those allocated to the usual care group may take up the experimental intervention or be dissatisfied with the usual care allocation and drop out.^11^ This ‘contamination’ effect might substantially dilute any treatment effect.^11^

We previously carried out a single-arm pilot study of a tailored exercise intervention in 37 MM survivors. The intervention was acceptable and feasible, and showed improvements in fatigue, QoL and muscle strength over 6 months.^12^ To confirm the beneficial effects of exercise training, we designed a randomised trial utilising a Zelen design that aimed to avoid contamination bias.^13^ The Zelen design has to our knowledge rarely been used in cancer exercise trials. Unlike previous RCTs in MM, our trial focused on patients after they finished first-line therapies, because this is usually the longest treatment-free interval. The primary aim was to explore the benefits of an individually tailored exercise programme on levels of fatigue. Secondary outcomes included QoL, fitness and strength.

## Methods

### Study design

This was a randomised parallel trial of an exercise intervention. The study was conducted at a tertiary hospital in central London (UK), and had full ethical approval by the National Research Ethics Service Committee London—Queens Square. The study protocol is available in Supplementary Information 1. The completed CONSORT and TIDieR checklists are available in Supplementary Information 2 and 3, respectively.

### Participants

MM survivors were recruited by clinicians at their routine myeloma appointments. They were eligible if they had stable disease for at least 6 weeks, completed their initial treatment or were on maintenance therapy, had ECOG performance status 0–2 and were able to undergo a regular exercise programme (exclusion criteria in Supplementary Table 1). Eligibility assessment was performed by a doctor, and included tests for disease status, X-rays, magnetic resonance imaging or electrocardiogram as clinically indicated. Scans and plain X-rays were reviewed in the multidisciplinary meeting and assessed for fracture risk using Mirels score.^14^ Patients interested to participate were referred to a researcher for more information. All participants provided written informed consent to participate.

### Randomisation and masking

The adapted Zelen study design with double consent aimed to avoid the potential contamination bias.^13^ Patients were invited to participate in an observational cohort study to understand the relationship between lifestyle, physical and mental health and biomarkers, without specific emphasis on exercise, as per the published protocol.^15^ The initial participant information sheet (PIS) outlined the types and timings of assessments to be performed, including all outcome measures. Following consent and the baseline assessment, participants were randomised with a 3:1 ratio to either the exercise or usual care, and all received care as usual. Randomisation was performed by R.J.B. or S.P. using minimisation (MinimPy software) stratified by gender and fatigue score (≤37 and >37).

After randomisation, R.J.B. or S.P., neither of whom had patient contact, immediately informed the physiotherapists (J.L. and O.M.) of the allocation by phone. The physiotherapists contacted only the participants who had been allocated to the exercise arm to invite them to participate in an interventional study of exercise, and to provide a second consent. The allocation was concealed from all other researchers. The second PIS contained only details specific to the exercise-training programme. Participants who agreed to the exercise intervention provided a second consent, whilst those who declined continued with the follow-up assessments as per the observational study. The usual care group were unaware that they had been randomised on any details about the intervention. Most assessments were performed by researchers (B.P., D.A.K. or M.H.) blinded to allocation, but occasionally performed by the unblinded physiotherapist (J.L.) given resource constraints.

### Procedures

Participants received a 6-month aerobic and resistance exercise-training programme individualised to their abilities, and based on published guidelines for cancer survivors.^16^ This aimed to ensure suitability, safety and adherence to the programme. For the first 3 months, the intervention involved one session per week at the hospital gym in central London, and participants were expected to exercise a further two times per week at home. They were provided with exercise diaries for completion. The physiotherapist reviewed the diaries at the following session, provided feedback on behaviour, facilitated goal setting and action planning and tailored the exercises accordingly. In the second 3 months (months 4–6), participants were expected to exercise at home three times per week and exercised at the hospital gym once per month.

Aerobic training consisted of treadmill walking, cycle ergometer, cross-trainer or stepper (whichever they preferred) at a target intensity of 50–75% of predicted maximum heart rate, calculated during baseline cardiorespiratory fitness testing. Target duration of aerobic training was increased progressively up to 30 min, in minimum 10-min bouts. Gradual progression was achieved by increasing exercise duration by 5 min and intensity by 5% maximum heart rate every 4 weeks. Resistance exercises covered the trunk, and upper and lower body, using weightlifting equipment, body weight or resistance bands. Resistance exercises were prescribed, individually tailored and gradually progressed by the study physiotherapist using 10-repetition maximum assessment, according to published principles.^17^ All sessions were delivered by a physiotherapist trained in behavioural support using Habit Theory,^18^ so that participants could create exercise habits outside the sessions (details in Supplementary Information 1).

The usual care group were asked to maintain their usual lifestyle. All outcomes were assessed at baseline, 3, 6 and 12 months.

### Outcome measures

The primary outcome was fatigue improvement at 3 months because it is considered a central symptom in multiple myeloma survivors, and our pilot study showed that exercise may improve fatigue.^12^ Fatigue is also a major issue for patients with solid cancers, and perhaps the most common endpoint (or co-endpoint) of trials of behavioural change or other non-drug interventions aimed at improving QoL/symptoms. It was measured by the Functional Assessment of Chronic Illness Therapy–Fatigue (FACIT-F) scale that has demonstrated reliability and sensitivity to change in cancer patients.^19^ Higher scores denote lower fatigue levels. Secondary QoL outcomes were the functional and emotional subscales of the Functional Assessment of Cancer Therapy—General instrument (FACT-G),^20^ and emotional distress using the Hospital Anxiety and Depression Scale.^21^

Cardiorespiratory fitness was assessed using an ergometer bike, and VO_2peak_ was estimated using the Metasoft Expair software according to US recommendations.^22^ Lower-limb muscle strength was assessed with each leg, ten-repetition maximum load and leg extension test, and averaged over both legs.^12^ Hand grip strength was measured with a handheld dynamometer. Three measurements were taken from each arm and averaged for analysis. Participants wore a triaxial accelerometer (ActiGraph-wGT3X-BT, Florida, USA) for 7 consecutive days above the non-dominant hip, and physical activity data were integrated into 60-s epochs and converted into total daily activity adjusted for wear time as mean accelerometer counts per minute.

As exercise can improve body composition, body weight (kg), percentage of body fat (%) and muscle mass (kg) were assessed using bioelectrical impedance (TANITA MC-980), which has been shown to have acceptable reliability and accuracy.^23^ Height was measured with a stadiometer with shoes removed, and the body mass index (BMI) was calculated.

Patients had regular haematology and biochemistry blood tests as per routine clinical care, and to confirm that their disease remained stable during the study. Patients with evidence of disease progression, whether biochemical or clinical, were withdrawn from the trial, because of the potential confounding effects of disease and treatment-related effects on outcome measures, and on adherence to the exercise regimen. Adverse events were monitored at each visit. The physiotherapist spoke to intervention participants prior to commencement of each exercise session to identify any potential adverse events.

### Statistical analysis

We aimed to detect an improvement in fatigue score of four units (FACIT-F) at 3 months, considered a clinically significant effect,^24^ equivalent to a standardised difference of 0.69. This was based on the observed 4.3-unit increase in the 6-month fatigue score and standard deviation 5.8 from our pilot study.^12^ With 80% power and two-sided 5% statistical significance, 34 patients per arm were required. To allow for patients declining allocation to the exercise arm, a randomisation ratio of 3:1 was used, aiming to randomise ~140 patients.

Analyses were performed for each variable using linear regression, with the baseline value and treatment group as covariates. The primary analysis was modified with intention to treat using available cases. It compared those who accepted the intervention with the usual care group. Thus, participants who declined the exercise programme were excluded. This method of analysis aimed to ensure that a high decline rate would not dilute the effect size, and that the data could form the basis for future larger trials.

Per-protocol analyses compared the usual care group with those who had high adherence to the group exercise sessions. The latter were defined as the participants attending at least 6 of the 12 (50%) sessions in the first 3 months, because training for less than 2 days per week appears insufficient for maximising muscle development.^25^

We examined the correlation between fatigue and other variables at baseline. A repeated measures/mixed-effect analysis was also performed for all time points up to 6 months. We undertook a planned per-protocol analysis of the subgroup with clinical fatigue at baseline (i.e. with a fatigue score below 34 as defined appropriate for cancer patients).^26^ No allowance was made for multiple testing, because we wanted to see which outcomes were improved to be considered for a subsequent larger trial. Imputation was not applied, because few patients had missing data at 3 or 6 months, and there were no striking differences between these patients and those who had non-missing data. SPSS (v25) was used for all analyses. The trial was prospectively registered at ISRCTN (ID:38480455).

We conducted semi-structured interviews in the exercise group to elicit their attitudes and experiences (*n* = 20). These results will be reported in detail in a separate paper, but a summary is provided here. Data were transcribed, and two researchers coded them in NVivo (v10) to identify data-driven themes using the six-stage thematic analysis at an explicit level with a realist approach.^27^

## Results

Between June 2014 and November 2016, 313 patients were identified, of whom 131 were randomised. Fifty-one of 89 patients (57%) allocated to the exercise programme accepted the intervention. Baseline characteristics were similar between the randomised subgroups of patients (Table 1). Most participants had bone disease (69%), around one-third reported pain and 22% were on maintenance treatment (thalidomide or lenalidomide) (Supplementary Table 2).

**Table 1:** Baseline characteristics of all participants.

Baseline characteristics	Control (*n* = 42)	Randomised to active intervention (*n* = 89)	Declined active intervention (*n* = 38)	Accepted active intervention (*n* = 51)	Completed active intervention (*n* = 41)
Age, median (range)	63 (40–80)	64 (35–86)	64 (36–86)	63 (35–86)	64 (41–86)
Female sex, *n* (%)	18 (43%)	41 (46%)	17 (45%)	24 (47%)	18 (49%)
*Ethnicity,* n *(%)*					
White	36 (86%)	74 (83%)	35 (92%)	29 (77%)	32 (78%)
Black	3 (7%)	9 (10%)	1 (3%)	8 (16%)	5 (12%)
Asian	3 (7%)	4 (5%)	1 (3%)	3 (6%)	3 (7%)
Other	0 (0%)	2 (2%)	1 (3%)	1 (2%)	1 (2%)
*Type of myeloma*					
IgG	27 (64%)	52 (58%)	22 (58%)	30 (59%)	24 (59%)
IgA	5 (12%)	15 (17%)	7 (18%)	8 (16%)	7 (17%)
Light chain	7 (17%)	17 (19%)	8 (21%)	9 (18%)	7 (17%)
Non-secretory/oligo-secretory	3 (7%)	5 (6%)	1 (3%)	4 (8%)	3 (7%)
*Autologous stem-cell transplantation*					
No	4 (10%)	14 (16%)	7 (18%)	7 (14%)	6 (15%)
Yes	38 (90%)	75 (84%)	31 (82%)	44 (86%)	35 (86%)
On maintenance treatment	4 (10%)	18 (20%)	8 (21%)	10 (20%)	10 (24%)
Bone disease	29 (69%)	61 (69%)	24 (63%)	37 (73%)	29 (71%)
Pain	13 (31%)	35 (39%)	15 (39%)	20 (39%)	16 (39%)
Prior surgery	11 (26%)	16 (18%)	7 (18%)	9 (18%)	8 (20%)
Radiotherapy	8 (19%)	23 (26%)	9 (24%)	14 (28%)	9 (22%)
Time since treatment, median (range), months	20 (2, 251)	14 (2, 161)	15 (2, 161)	13 (2, 138)	12 (2, 84)
*ECOG performance score*					
0	33 (79%)	70 (79%)	31 (82%)	39 (76%)	31 (76%)
1	9 (21%)	19 (21%)	7 (18%)	12 (24%)	10 (24%)

### Retention and adherence

**Fig. 1:**
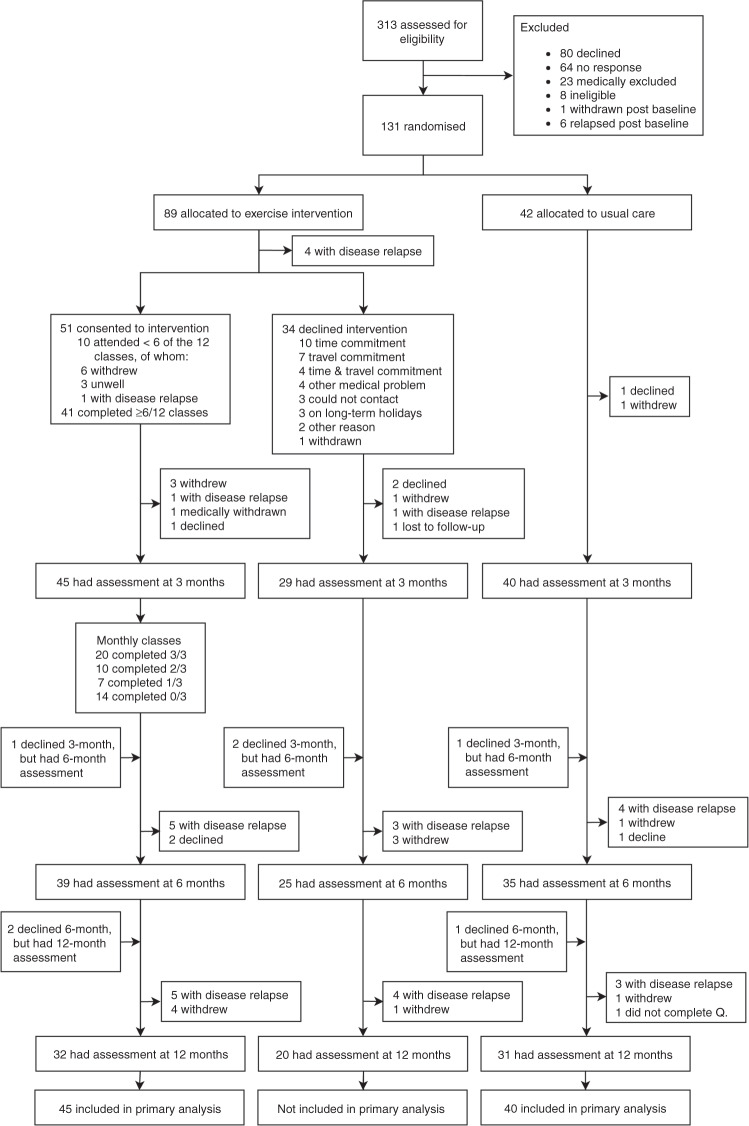
CONSORT flow diagram. The diagram displays the progress of the participants through the MASCOT trial. Q: Questionnaire.

Retention rates at 3 months were high: 88% for those accepting the intervention, 76% for those declining the intervention and 95% for the usual care group. At 6 months, these rates were 76%, 66 and 83%, respectively (CONSORT diagram in Fig. 1). The participants in the exercise group (*n* = 51) attended a median of 9 out of 12 exercise classes (75%; range: 1–12), and 41 (80%) participants attended at least 50% of the 12 classes in the first 3 months. The reasons for non-attendance are shown in Fig. 1. Between months 4 and 6, 20 participants (64.5%) completed all three-monthly classes.

### Correlation analysis at baseline

Correlation analysis showed that fatigue at baseline was not correlated with age (*r* = −0.09, *p* = 0.41) or time since treatment (*r* = 0.15, *p* = 0.14), but with measures of physical fitness. Thus, participants reporting more fatigue had higher body fat percentage (*r* = 0.29, *p* = 0.005), and lower VO_2peak_ (*r* = −0.33, *p* < 0.001), and leg strength (*r* = −0.25, *p* = 0.017).

### Primary (modified intention-to-treat) analysis

There was little effect of the exercise programme on fatigue at either 3 (between-group difference 1.6 units) or 6 months (difference 0.3 units) (Tables 2 and 3). From the repeated measures analysis, the mean difference between the groups was 0.8 (95% CI: −3.0 to 4.7) for time points up to 6 months. There was no evidence of between-group differences in changes in physical or emotional functioning, anxiety or depression at 3 or 6 months (Tables 2 and 3).

**Table 2:** Primary and secondary outcome measures at 3 months among randomised controls and those patients who agreed to the exercise program and treatment effects (mean difference between the groups, adjusted for baseline values).

	Exercise			Control			Mean difference (95% CI)	*P*-value
	Baseline	3-month	*N*	Baseline	3-month	*N*		
*Quality of life*								
Fatigue	39.6 (9.3)	40.9 (9.8)	45	41.1 (10.8)	40.9 (9.9)	40	1.6 (−1.1, 4.3)	0.25
FACT—functional	19.7 (6.7)	20.1 (6.1)	45	21.4 (5.1)	21.8 (6.0)	40	0.0 (−1.5, 1.5)	0.97
FACT—emotional	19.8 (3.9)	19.6 (3.5)	45	19.5 (3.5)	19.5 (4.4)	40	0.0 (−1.5, 1.5)	0.99
HADS anxiety	4.7 (3.5)	4.3 (3.6)	42	5.4 (3.2)	5.0 (3.3)	39	–0.4 (−1.6, 0.8)	0.49
HADS depression	3.5 (2.7)	3.2 (2.5)	44	3.3 (2.6)	3.5 (3.9)	40	−0.6 (−1.7, 0.5)	0.26
*Anthropometry*								
% Fat	30.5 (8.5)	28.7 (8.3)	43	30.9 (10.2)	31.0 (10.8)	38	−0.9 (−1.9, 0.1)	0.07
Muscle mass (kg)	50.5 (10.9)	51.2 (10.0)	43	52.0 (10.3)	52.3 (10.4)	38	0.4 (−0.3, 1.0)	0.23
Weight (kg)	76.9 (15.2)	76.1 (14.8)	44	80.7 (17.6)	81.4 (18.1)	39	–0.4 (−1.3, 0.6)	0.43
*PA and fitness*								
PA (counts per minute)	312.4 (95.1)	326.5 (96.7)	39	353.5 (87.3)	342.8 (74.7)	30	10.3 (–22.1, 42.6)	0.53
Leg muscle strength (kg)	44.3 (26.0)	63.3 (23.3)	40	53.3 (22.1)	61.1 (19.1)	34	8.4 (0.5, 16.3)	0.04
Grip strength (kg)	28.1 (11.1)	29.4 (9.9)	43	31.3 (10.0)	31.7 (9.8)	37	0.7 (–1.0, 2.4)	0.42
VO_2_ peak (ml/kg/min)	18.1 (6.9)	20.1 (6.9)	40	19.3 (7.3)	19.8 (7.1)	37	1.5 (−0.2, 3.2)	0.08

*PA* physical activity.

Fatigue is on a scale 0–52 (high scores mean less fatigue). FACT—functional is on a scale 0–28, FACT—emotional is on a scale 0–24 and HADS—anxiety and depression are each on a scale 0–21 (high scores mean better QoL).

**Table 3:** Primary and secondary outcome measures at 6 months among randomised controls and those patients who agreed to the exercise program and treatment effects (mean difference between the groups, adjusted for baseline values).

	Exercise			Control			Mean difference (95% CI)	*P*-value
	Baseline	6-month	*N*	Baseline	6-month	*N*		
*Quality of life*								
Fatigue	39.8 (8.7)	41.1 (9.1)	39	43.4 (8.1)	43.7 (8.4)	35	0.3 (–2.6, 3.1)	0.85
FACT—functional	19.9 (6.7)	20.6 (5.4)	38	22.6 (4.2)	21.3 (6.0)	35	0.1 (–2.4, 2.7)	0.91
FACT—emotional	19.6 (4.1)	19.5 (4.1)	38	19.9 (3.3)	20.1 (3.5)	35	−0.3 (−1.6, 0.9)	0.59
HADS anxiety	4.9 (3.6)	5.3 (3.9)	39	5.2 (3·0)	4.4 (2.9)	33	1.1 (0.0, 2.1)	0.05
HADS depression	3.6 (2.8)	2.8 (2.4)	38	2.9 (2.4)	2.7 (2.5)	35	–0.3 (−1.2, 0.7)	0.57
*Anthropometry*								
% Fat	28.5 (8.5)	28.6 (8.0)	34	30.0 (10.7)	29.7 (10.8)	34	0.4 (−0.6, 1·4)	0.47
Muscle mass (kg)	51.1 (10.2)	51.6 (9.8)	34	53.1 (10.8)	53.4 (10.6)	34	–0.1 (–0.8, 0.7)	0.84
Weight (kg)	76.0 (14.8)	76.4 (14.3)	35	81.5 (19.0)	81.5 (18.7)	34	0.2 (−1.0, 1.3)	0.77
*PA and fitness*								
PA (counts per minute)	314.2 (92.9)	301.8 (79.2)	32	352.8 (89.2)	342.3 (103.9)	29	−12.4 (−45.9, 21.1)	0.13
Leg muscle strength (kg)	44.8 (25.3)	67.4 (23.9)	33	58.6 (20.6)	61.8 (23.4)	31	10.8 (1.2, 20.5)	0.03
Grip strength (kg)	28.7 (10.0)	30.1 (10.0)	34	33.2 (9.5)	34.0 (9.0)	34	0.5 (−1.3, 2.3)	0.58
VO_2_ peak (ml/kg/min)	18.7 (7.6)	19.5 (6.2)	32	20.7 (7.4)	19.4 (7.8)	31	1.2 (−1.0, 3.5)	0.27

*PA* physical activity.

Fatigue is on a scale 0–52 (high scores mean less fatigue). FACT—functional is on a scale 0–28, FACT—emotional is on a scale 0–24 and HADS—anxiety and depression are each on a scale 0–21 (high scores mean better QoL).

Leg muscle strength was significantly improved following the exercise intervention. The between-group improvement was 8.4 kg (95% CI: 0.5–16.3) in favour of exercise at 3 months, and 10.8 kg (95% CI: 1.2–20.5) at 6 months (Tables 2 and 3). From the repeated measures model, there was strong evidence that the difference in leg strength between groups depended on the time point (*p* < 0.002 for the interaction, Supplementary Fig. 1).

Only 14.6% of those in the intervention group and 15.8% in the usual care group were meeting the 150-min/week moderate-to-vigorous physical activity guidelines at baseline. Baseline obesity (BMI ≥ 30 kg/m^2^) was seen in 27 and 38% of participants in the exercise and usual care group, respectively. No effect of the intervention on physical activity was observed. There seemed to be a small improvement in percentage of body fat (−0.9%) at 3 months (*p* = 0.07), which was not seen at 6 months (*p* = 0.47) in the exercise group. VO_2peak_ also showed a trend to improvement at 3 months (*p* = 0.08), but this was not seen at 6 months (*p* = 0.27) (Tables 2 and 3). There were no between-group differences for grip strength at 3 or 6 months (Tables 2 and 3).

Supplementary Tables 3–5 compare the characteristics of patients who went through the exercise programme, and those who declined it after being randomised. With the exception of leg strength at 3 and 12 months, there were no striking differences between the groups.

### Secondary (per-protocol) analysis

**Fig. 2:**
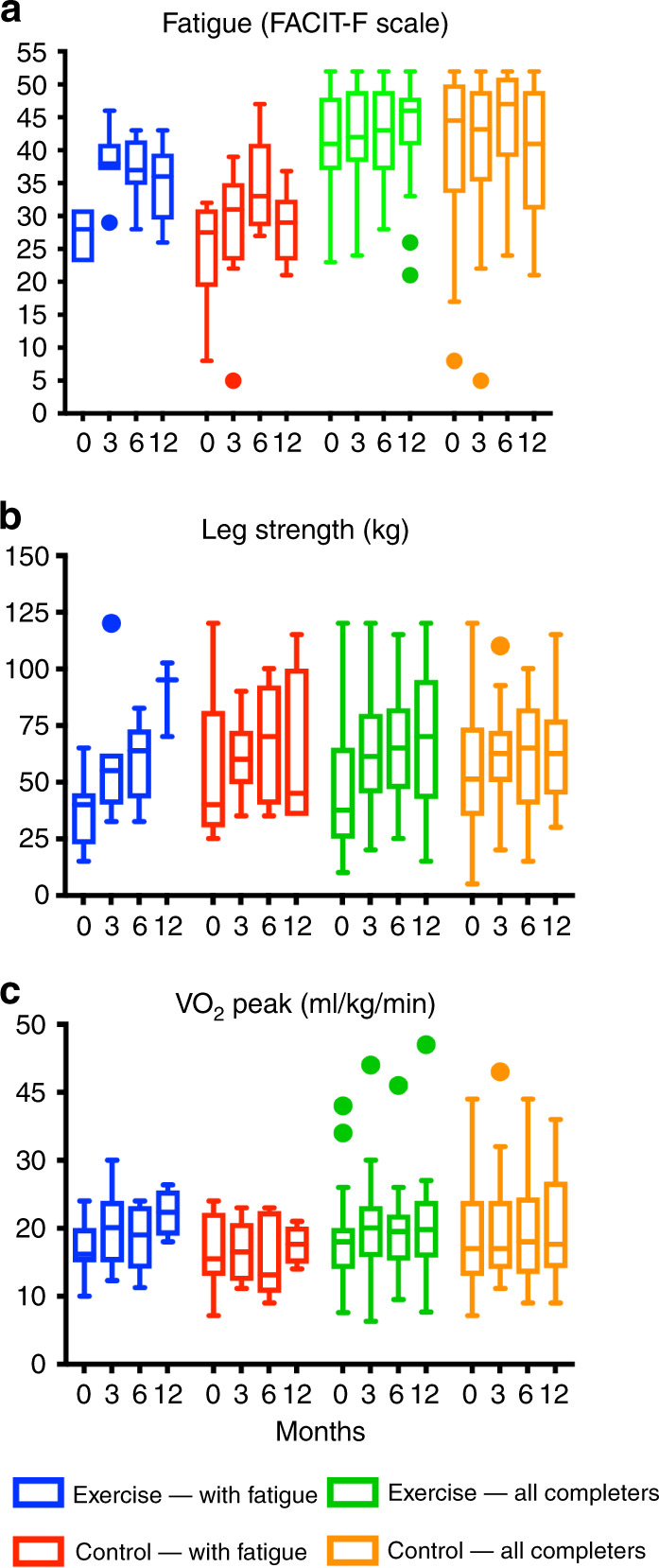
Tukey plots. Tukey plots for fatigue (**a**), leg strength (**b**) and VO_2_ peak (**c**) for participants with clinical fatigue at baseline (FACIT-F score < 34), and all participants in the per-protocol analysis (had high adherence to the exercise programme) at each time point.

Figure 2 and Supplementary Tables 6–7 show the per-protocol analysis (patients in the exercise group who attended at least 50% of the sessions) for several endpoints. The results were broadly similar to the primary analysis, except for a significant positive effect on VO_2peak_ (+1.2 ml/kg/min, *p* = 0.02) and on percentage of body fat (−0.9%, *p* = 0.05, Supplementary Table 6). The benefits on leg strength were also seen in this analysis: differences of 7.9 kg (*p* = 0.05) and 11.3 kg (*p* = 0.03) at 3 and 6 months, respectively.

### Long-term effects

Looking at the longer-term effects, Supplementary Table 5 presents descriptive data at 12 months. The means of the outcome measures in each trial group were consistent with those at 3 and 6 months. The increase in leg muscle strength in the modified ITT exercise group was maintained at 12 months, suggesting some longer-term benefit of the intervention (Fig. 2b; Supplementary Table 5). There was no long-term impact on fatigue or other measures.

### Exploratory analysis

At baseline, 10 (19%) and 10 (24%) participants in the exercise and usual care groups respectively, had clinical fatigue. Therefore, we undertook a post hoc exploratory subgroup analysis of those with clinical fatigue who had completed ≥50 of classes (exercise: *n* = 7, usual care: *n* = 10), because there could be more scope to see benefits in this particular group. Compared with the whole cohort, 17 patients with clinical fatigue at baseline had worse ECOG scores (35% had ECOG 0 compared with 79% in all patients), more pain (71% vs. 37%) and higher incidence of previous surgery (41% vs. 21%) as shown in Supplementary Table 2.

Figure 2 shows fatigue, leg strength and fitness level (VO_2peak_) in patients with baseline clinical fatigue, compared with the group as a whole. Patients with clinical fatigue who exercised improved their fatigue scores at 3 months (from 27.7 ± 3.6 to 38.4 ± 5.1), and this was maintained at 6 and 12 months (37.2 ± 5.2 and 34.8 ± 6.1). However, improvements were also seen in the usual care group (from 25.0 ± 7.8 at baseline to 28.1 ± 10.8 at 3 months, 34.7 ± 7.6 at 6 months and 28.5 ± 5.7 at 12 months, Supplementary Table 8). In a repeated measures analysis (baseline up to 6 months), the mean between-group difference in fatigue score was 6.3 (95% CI: −0.6 to 13.3, *p* = 0.07). There was a positive trend in leg strength in the exercise group over time (Fig. 2b). However, leg strength was not significantly different between groups (repeated measures’ mean difference from usual care: −2.5 kg (95% CI: −26.0 to 20.9)). There was no change in VO_2peak_ in the exercise group up to 6 months, and no material effects on other endpoints (Supplementary Fig. 2).

### Adverse events

No serious adverse events were reported by study participants. One participant reported hip pain while performing the home-exercise programme that was spontaneously resolved. Four participants reported lower back pain during the intervention period, but it was unclear if this was related to exercise. Haematology parameters stayed stable throughout the study (Supplementary Table 9).

### Qualitative feedback

Participants’ feedback showed that the main reasons for declining the intervention were time and travel constraints. Qualitative interviews helped in understanding the less tangible, yet equally important, benefits. Box 1 shows sample quotes (details in a separate paper). Participants were generally pleased to participate, appreciated the opportunity to improve their physical well-being and reported increased confidence in exercising because of professional supervision. Patients also felt fulfilled and described a sense of achievement. Most of them found the level of exercise appropriate and maintainable, but reported travelling as the main attendance barrier.

Box 1: Participants’ experience of the exercise intervention from interviews

**Table:** 

Participants’ experience	Key quotes
Enjoyment and self-confidence	*“I think the biggest thing has been for me the confidence to know it’s okay to do exercise. I was frightened to exercise before, I was frightened that I was going do some damage because I’d done so much damage to my bones. So, the biggest thing was having the confidence instilled in me that I can do it. You certainly wouldn’t have got that through being given a fact sheet about what’s possible. Having a really highly skilled physio supporting me physically, mentally and emotionally through that was brilliant. It really gave me the confidence and ability to go out and have a more active life really.”*
Sense of fulfilment and achievement	*“I felt fulfilled, motivated. That’s always nice to think, ‘I’ve actually achieved something’.”*
	*“I like the sense of achievement after, I like the sense of wellbeing because you feel so alive and engaged and I have a little twinkle.”*
Factors influencing intervention adherence	*“You track that [i.e. Borg Rating of Perceived Exertion] so that as you come into each session, you’re – you know, Jo would have a look and say, ‘Ah. Anything feel a bit easy? That one looks – yeah, that one feels a bit easy. Right, well, we’ll increase the difficulty there.’ Or actually, ‘You’re right up at the limit there. Let’s just keep trying to build up to that one.’ You know, so we’re constantly checking each exercise in terms of was it stretching you enough um and you know, do we need to make it any – any adaptations to help you build up? So, there’s a few things I had to have adapted so that I could build up to it’cause they were too difficult, and you know, I was able to progress, and other things that you know, felt quite easy so we increased the difficulty of those. But it was you know, very, very personalized every week, so that was great.”*
	*“The only thing is that it takes quite a long time, you know, to arrive here, the exercise and so on. Basically, it’s half of your day gone.”*

## Discussion

MASCOT was the first RCT to evaluate the benefits of an exercise intervention in MM survivors who have completed treatment. We observed significant improvements in leg muscle strength at 3 and 6 months that remained at 12 months, but little effect on fatigue, the primary outcome. Post hoc analysis showed that participants with clinical levels of fatigue reported some improvement in fatigue following the intervention. The pain and bone morbidity seen at baseline in many patients would theoretically make them unlikely candidates for exercise programmes. However, adherence was good, and uptake was comparable to other exercise studies.^28^ The intervention was safe, and no notable adverse events were observed. The Zelen design aimed to avoid contamination bias, thereby producing more reliable estimates of effect than other cancer trials of exercise. MASCOT followed on from our single-arm trial, in which fatigue improved by 4 points at 6 months.^12^ A likely reason for the smaller effect on fatigue in MASCOT is that patients had less fatigue at baseline than in the pilot study (mean score 40.7 vs. 37.4), hence less scope for improvement. This is partly supported by our exploratory analyses, suggesting improvement in fatigue in the subset of patients with clinical fatigue at baseline, along with increased leg strength. However, future larger trials in patients with clinical fatigue are required.

The two previous RCTs of exercise in MM patients involved home-exercise programmes during high-dose chemotherapy and autologous stem-cell transplantation.^9,10^ They were inconclusive and had a short follow-up. One had only 24 patients, though the results suggested improvements in muscle strength (in line with our data) and sleep. The other study (187 patients) reported no effect on fatigue, but did not measure strength/physical fitness, nor analysed subgroups according to baseline fatigue score. Our study was designed to improve upon these limitations, with a tailored and supervised programme (once-weekly gym attendance), individualised progression of intensity and accompanied by behavioural support using Habit theory. We also aimed to reduce contamination through a blinded control arm, utilising a modified Zelen design. Further strengths of our MASCOT study include timing of intervention after treatment to reduce the risk of fracture and increase the likelihood of participants completing the strength tests, the use of a theory-based intervention, comprehensive objective outcomes, a more ethnically diverse sample than previous trials and high adherence to the exercise sessions.

The limitations of the current trial include the low levels of the ECOG score at baseline. Despite being open to patients with ECOG scores of 0–2, all patients in our study had ECOG 0–1. Furthermore, the mean fatigue score of 37.4 was substantially higher, indicating less fatigue, compared with a previous study of 88 MM survivors that reported a mean FACIT-F-fatigue score of 20.2.^7^ We were recruiting from a central London tertiary centre where patients are referred for autologous stem-cell transplantation, and the majority of our cohort were in their first line of treatment. Such patients often have higher functioning and QoL than those in subsequent treatment phases.^29^ Hence, there was less room for improvement in some of the outcomes we studied.

Even though the exercise programme involved weekly attendance at a central gym, the uptake (57%) was consistent with a systematic review of 65 cancer exercise trials using a standard RCT design where uptake was estimated at 63% (range 33–80%).^28^ At 6 months, 76% remained in the programme. Of the decliners, 62% were interested in participating in the intervention, but were not keen on the extra time/travel commitment. This highlights the need to design and test exercise programmes with better access and less burden for patients.

The modified intention-to-treat analysis was used as the decline rate within the intervention group may otherwise have diluted any potential intervention effect. Furthermore, we did not generally observe differences between those who accepted and declined the intervention. Thus, the modified intention-to-treat analysis allowed us to explore whether the intervention had an effect on the outcomes under study.

Our findings of increased leg muscle strength are particularly relevant to an older group of cancer survivors with osteolytic bone disease. These were clinically large effects, which could be expected to help improve patients’ general mobility, as well as providing some lessening of physical fatigue. For MM survivors, the risk of falls related to advanced age is increased by deconditioning, resulting from bone morbidity and debilitating effects of chemotherapy, and the fracture risk is high. Increased muscle strength reduces the risk of falls; thus, this in itself is an important benefit of physical exercise. Our preliminary results for fatigue reflect those of two studies of exercise intervention in patients undergoing stem-cell transplantation (any haematological cancer), which reported no improvement in fatigue,^30,31^ though one showed better physical functioning.^30^ A third trial in haematological cancer survivors (37 patients) indicated a large benefit of exercise (post cancer treatment) on fatigue at 3 months; however, with a different tool (Schwartz Cancer Fatigue scale), benefits were lost by 6 months, and only four patients had MM.^32^

## Conclusion

Our RCT in MM survivors demonstrated that a tailored exercise programme was safe, improved muscle strength in all patients and indicated preliminary evidence of improved fatigue in those who had clinical fatigue at baseline. Multiple myeloma survivors could consider structured exercise programmes, and clinicians should be encouraged to offer or refer patients for exercise support. Larger trials should now focus on patients with clinical fatigue, and modify the intervention delivery format to reduce barriers to recruitment and attendance providing more support for home-based activities.

## Supplementary information

Supplementary Information 1

Supplementary Information 2

Supplementary Information 3

## Data Availability

The data sets used and/or analysed during the current study are available from the corresponding author on reasonable request.
