# Supplementary material for: Fatigue, quality of life and physical fitness following an exercise intervention in multiple myeloma survivors (MASCOT): an exploratory randomised Phase 2 trial utilising a modified Zelen design
Source: Br J Cancer. 2020 May 21;123(2):187–95. doi: 10.1038/s41416-020-0866-y (PMC7374110; doi:10.1038/s41416-020-0866-y)
Supplement: Supplementary file 1 — Supplementary Information 1 [file 41416_2020_866_MOESM1_ESM.pdf]

Supplementary Information 1

Details on ethics approval

The study protocol and documents have been reviewed and approved by the relevant sponsor and National Research Ethics Service Committee London - Queen Square (Reference: 13/LO/1105, 18 December 2013).

Behavioral support during the exercise intervention

The behavioral change techniques included pros and cons, instructions on how to perform the behavior, behavioral practice/rehearsal, demonstration of behavior, feedback on behavior, social reward, habit formation, behavioral self-monitoring, behavioral goal setting with the SMART (specific, measurable, achievable, relevant, and time-specific) principle, action planning, feedback on behavior, mental rehearsal of successful performance, problem solving, restructuring the physical environment, adding objects to the environment, and social support (unspecified). Stress management techniques were also taught during the eighth session.

Table 1: Exclusion criteria in the MASCOT study

Exclusion criteria
1. Spinal instability (as assessed on radiology in multi-disciplinary team meetings)
2. Recent (within 4 weeks) spinal or other surgery for pathological fractures
3. Abnormal resting electrocardiogram, where clinically indicated unexplained by further cardiological work-up
4. At risk of pathological fracture based on Mirel's score [29]
5. Current participation in an exercise program as part of a research study
6. Unstable angina
7. Musculoskeletal disease limiting mobility
8. Cognitive impairment that impedes ability to complete questionnaires

Table 2 Baseline characteristics of the whole cohort and the participants with clinical fatigue in the sensitivity analysis

Baseline characteristics	All trial participants (n=131)	Participants with clinical fatigue in the sensitivity analysis (n=17)
Age, median (range)	63 (35, 86)	59 (41, 86)
Female sex, n (%)	59 (45%)	7 (41%)
Ethnicity, n (%)		
White	110 (84%)	11 (65%)
Black	12 (9%)	2 (12%)
Asian	7 (5)	4 (24%)
Other	2 (2)	0 (0%)
Type of myeloma		
IgG	79 (60%)	7 (41%)
IgA	20 (15%)	3 (18%)
Light Chain	24 (18%)	4 (24%)
Non-secretory/Oligo-secretory	8 (6%)	3 (18%)
ASCT	113 (87%)	13 (77%)
On maintenance treatment	29 (22%)	3 (18%)
Bone disease	90 (69%)	13 (77%)
Pain	38 (37%)	12 (71%)
Prior surgery	27 (21%)	7 (41%)
Radiotherapy	31 (24%)	6 (35%)
Time since treatment, median (range), months	28 (0, 251)	13 (2, 251)
ECOG Performance score		
0	103 (79%)	6 (35%)
1	28 (21%)	11 (65%)

Table 3 Mean ± SD of outcome measures at 3-months for intervention and control participants

Outcome measure	Accepted intervention		Declined intervention		Control	
	N	Mean ± SD	N	Mean ± SD	N	Mean ± SD
Fatigue	45	40.9 ± 9.8	29	37.6 ± 16.2	40	40.9 ± 9.9
FACT-Physical	45	20.1 ± 6.1	29	20.4 ± 7.5	40	21.8 ± 6.0
FACT-Emotional	45	19.6 ± 3.5	29	19.0 ± 4.3	40	19.5 ± 4.3
HADS Anxiety	42	4.3 ± 3.6	29	4.8 ± 3.1	40	5.0 ± 3.3
HADS Depression	44	3.2 ± 2.5	29	3.3 ± 3.5	40	3.5 ± 3.9
% Fat	44	28.7 ± 8.3	26	29.9 ± 7.2	38	31.0 ± 10.8
Muscle mass (kg)	44	51.2 ± 10.0	26	51.4 ± 10.2	38	52.3 ± 10.4
BMI (kg/m^2)	44	26.8 ± 4.7	26	27.5 ± 4.1	38	28.9 ± 6.1
Weight (kg)	44	76.1 ± 14.8	27	77.8 ± 14.3	39	81.4 ± 18.1
Leg muscle strength (kg)	41	63.3 ± 23.3	25	52.0 ± 26.4	35	61.1 ± 19.1
Grip strength (kg)	44	29.4 ± 9.9	27	29.5 ± 11.3	38	31.7 ± 9.8
Total PA (mean cpm)	42	322.5 ± 98.1	23	293.4 ± 81.5	37	341.2 ± 71.7
VO_2 peak (ml/kg/min)	42	20.1 ± 6.9	25	19.9 ± 10.8	39	19.8 ± 7.1
Diastolic blood pressure (mmHg)	44	79.7 ± 12.0	27	79.4 ± 14.0	37	81.8 ± 10.9
Systolic blood pressure (mmHg)	44	134.4 ± 19.1	27	131 ± 16.1	37	137.1 ± 20.1
C-reactive protein	45	3.0 ± 4.0	28	4.6 ± 10.8	38	3.4 ± 4.2
IgA	45	1.3 ± 1.1	28	1.9 ± 4.2	38	1.4 ± 0.9
IgG	45	11.7 ± 6.1	28	15.4 ± 18.5	38	11.9 ± 5.7
IgM	45	0.5 ± 0.3	28	0.6 ± 0.4	38	0.7 ± 0.5
Hemoglobin	45	128.8 ± 15.8	28	127.9 ± 15.5	39	133.7 ± 12.6

cpm: counts per minute.

Table 4 Mean ± SD of outcome measures at 6-months for intervention and control participants

Outcome measure	Accepted intervention		Declined intervention		Control	
	N	Mean ± SD	N	Mean ± SD	N	Mean ± SD
Fatigue	39	41.1 ± 9.1	25	43.9 ± 8.9	35	43.7 ± 8.4
FACT-Physical	38	20.6 ± 5.4	25	21.7 ± 5.0	35	21.3 ± 6.0
FACT-Emotional	38	19.5 ± 4.1	25	20.4 ± 2.6	35	20.1 ± 3.5
HADS Anxiety	39	5.3 ± 3.9	25	4.0 ± 3.8	34	4.4 ± 2.9
HADS Depression	38	2.8 ± 2.4	25	2.8 ± 2.6	35	2.7 ± 2.5
% Fat	35	28.6 ± 8.0	22	28.9 ± 6.9	34	29.7 ± 10.8
Muscle mass (kg)	35	51.6 ± 9.8	22	51.0 ± 10.2	34	53.4 ± 10.6
BMI (kg/m^2)	35	27.0 ± 4.4	23	26.7 ± 3.7	34	28.6 ± 6.2
Weight (kg)	35	76.4 ± 14.3	23	76.1 ± 13.9	34	81.5 ± 18.7
Leg muscle strength (kg)	35	67.5 ± 23.8	22	58.4 ± 25.4	33	61.8 ± 23.4
Grip strength (kg)	35	30.1 ± 10.0	23	28.6 ± 12.3	34	34.0 ± 9.0
Total PA (mean cpm)	35	311.0 ± 84.5	24	325.3 ± 110.8	34	354.2 ± 102.8
VO_2 peak (ml/kg/min)	34	19.5 ± 6.2	21	20.1 ± 9.2	33	19.6 ± 7.8
Diastolic blood pressure (mmHg)	35	81.1 ± 12.6	23	73.4 ± 12.4	34	79.3 ± 11.9
Systolic blood pressure (mmHg)	35	136.9 ± 23.7	23	124.8 ± 14.3	34	133.2 ± 17.2
C-reactive protein	34	3.5 ± 4.9	22	8.9 ± 28.3	34	6.2 ± 11.2
IgA	35	1.5 ± 1.2	23	2.4 ± 5.4	34	1.7 ± 1.9
IgG	35	12.4 ± 5.8	23	10.9 ± 6.0	34	11.9 ± 6.6
IgM	35	0.6 ± 0.4	23	0.6 ± 0.4	34	0.7 ± 0.6
Hemoglobin	35	96.7 ± 54.6	23	104.3 ± 51.7	33	90.0 ± 59.8

cpm: counts per minute.

Table 5 Mean ± SD of outcome measures at 12-months for intervention and control participants

Outcome measure	Accepted intervention		High adherence to intervention		Declined intervention		Control	
	N	Mean ± SD	N	Mean ± SD	N	Mean ± SD	N	Mean ± SD
Fatigue	32	42.4 ± 9.1	30	43.4 ± 7.4	20	44.0 ± 8.0	31	40.5 ± 10.2
FACT-Physical	31	23.1 ± 5.3	29	23.5 ± 5.2	20	23.3 ± 8.7	31	21.2 ± 7.3
FACT-Emotional	31	21.1 ± 2.9	29	21.4 ± 2.4	20	20.8 ± 3.0	31	19.7 ± 4.0
HADS Anxiety	32	3.7 ± 3.2	30	3.4 ± 3.1	19	4.5 ± 4.2	30	5.0 ± 3.2
HADS Depression	32	2.5 ± 2.3	30	2.4 ± 2.3	20	2.8 ± 3.0	30	3.6 ± 3.3
% Fat	28	29.4 ± 6.7	26	29.8 ± 6.8	18	30.4 ± 6.9	28	31.5 ± 10.2
Muscle mass (kg)	28	50.4 ± 10.2	26	50.0 ± 9.9	18	50.1 ± 11.2	28	53.2 ± 10.3
BMI (kg/m^2)	28	26.8 ± 4.2	26	26.9 ± 4.2	18	26.9 ± 4.2	28	29.8 ± 6.5
Weight (kg)	28	79.4 ± 24.4	26	79.4 ± 24.7	19	86.5 ± 31.1	28	84.0 ± 19.8
Leg muscle strength (kg)	24	66.8 ± 31.4	23	68.4 ± 31.1	18	49.8 ± 21.0	26	63.2 ± 22.9
Grip strength (kg)	28	31.1 ± 12.1	26	31.3 ± 11.9	19	28.5 ± 12.8	27	40.7 ± 27.7
Total PA (mean cpm)	32	309.8 ± 93.1	28	304.2 ± 88.5	20	335.3 ± 106.8	28	334.9 ± 86.8
VO_2 peak (ml/kg/min)	24	20.7 ± 7.3	27	20.2 ± 7.5	18	19.8 ± 8.8	24	20.8 ± 7.7
Diastolic blood pressure (mmHg)	27	81.3 ± 11.2	25	81.1 ± 11.5	19	74.9 ± 13.4	28	82.6 ± 12.2
Systolic blood pressure (mmHg)	27	138.4 ± 22.7	25	138.1 ± 23.5	19	124.1 ± 15.8	28	135.1 ± 16.0
C-reactive protein	25	2.4 ± 2.0	23	2.4 ± 2.0	14	3.1 ± 2.9	27	3.5 ± 3.1
IgA	28	1.6 ± 1.2	26	1.6 ± 1.3	19	1.3 ± 0.7	28	2.6 ± 4.3
IgG	28	12.0 ± 4.9	26	12.1 ± 5.1	19	12.3 ± 5.7	28	11.5 ± 7.6
IgM	28	0.6 ± 0.4	26	0.6 ± 0.4	19	0.7 ± 0.4	27	0.8 ± 0.6
Hemoglobin	31	94.3 ± 59.3	29	96.0 ± 58.8	19	97.8 ± 54.6	29	99.5 ± 55.8

cpm: counts per minute

Table 6 Per protocol analysis: Primary and secondary outcome measures at 3 months among randomized controls and those patients who highly adhered to the exercise program, and treatment effects (mean difference between the groups, adjusted for baseline values)

	Exercise Mean (SD)			Control Mean (SD)			3-month adjusted treatment difference (95% CI)	P-value
	Baseline	3-month	N	Baseline	3-month	N		
Fatigue	40.8 (7.8)	42.5 (7.0)	40	41.7 (10.7)	40.9 (9.9)	40	2.1 (-0.5, 4.8)	0.11
FACT-Functional	19.6 (7.2)	20.4 (6.1)	40	21.7 (5.1)	21.8 (6.0)	40	0.3 (-1.2, 1.8)	0.69
FACT-Emotional	20.1 (3.1)	19.9 (3.4)	40	19.7 (3.5)	19.5 (4.3)	40	0.2 (-1.3, 1.8)	0.79
HADS Anxiety	4.5 (3.2)	4.2 (3.3)	39	5.3 (3.3)	5.2 (3.2)	39	-0.5 (-1.7, 0.7)	0.45
HADS Depression	3.4 (2.5)	2.9 (2.3)	40	3.1 (2.6)	3.5 (3.9)	40	-0.8 (-1.9, 0.4)	0.19
% Fat	30.1 (7.9)	29.4 (8.1)	39	30.9 (10.5)	31.0 (10.8)	38	-0.9 (-1.7, 0.0)	0.05
Muscle mass (kg)	50.1 (10.1)	50.8 (9.9)	39	52.2 (10.5)	52.3 (10.4)	38	0.5 (-0.1, 1.2)	0.10
Weight (kg)	76.1 (15.0)	76.4 (14.7)	40	80.9 (17.8)	81.4 (18.1)	39	-0.2 (-1.1, 0.7)	0.66
Total PA (mean counts per minute)	302.6 (90.7)	323.7 (100.0)	36	353.5 (87.3)	342.8 (74.7)	30	16.8 (-16.3, 49.8)	0.31
Leg muscle strength (kg)	43.5 (24.5)	61.3 (21.8)	37	56.0 (20.6)	60.6 (19.1)	34	7.9 (-0.1, 15.8)	0.05
Grip strength (kg)	27.9 (9.7)	29.2 (9.8)	39	31.6 (10.2)	32.0 (9.8)	37	0.6 (-1.1, 2.4)	0.49
VO_{2peak} (ml/kg/min)	17.6 (5.7)	20.0 (6.9)	36	19.7 (7.4)	20.0 (7.2)	37	1.2 (0.3, 3.7)	0.02
Systolic BP (mmHg)	136.1 (19.8)	134.6 (19.9)	38	137.6 (19.0)	137.1 (20.4)	36	-1.1 (-6.2, 4.0)	0.67
Diastolic BP (mmHg)	83.6 (12.3)	79.5 (12.3)	38	83.5 (12.1)	81.8 (11.0)	36	-2.4 (-5.9, 1.0)	0.16
C-RP (mg/L)	3.9 (4.8)	2.9 (3.9)	39	3.7 (4.3)	3.4 (4.2)	38	-0.5 (-2.2, 1.1)	0.51
IgA (g/L)	1.4 (1.1)	1.4 (1.1)	39	1.3 (0.8)	1.4 (0.9)	38	-0.1 (-0.2, 0.1)	0.21
IgG (g/L)	11.2 (4.9)	11.5 (5.3)	39	11.6 (5.1)	11.9 (5.7)	38	-0.1 (-1.2, 1.1)	0.88
IgM (g/L)	0.5 (0.3)	0.5 (0.3)	39	0.7 (0.4)	0.7 (0.5)	38	0.0 (-0.1, 0.1)	0.76
Hemoglobin (g/L)	128.4 (15.0)	129.9 (14.5)	38	132.7 (13.4)	133.5 (12.7)	38	-0.1 (-3.5, 3.3)	0.95

FACT: Functional Assessment of Cancer Therapy, HADS: Hospital Anxiety and Depression Scale, PA: Physical activity, CI: Confidence interval. Higher scores of fatigue indicate lower fatigue levels. Higher scores in the functional, emotional, anxiety, and depression scales indicate higher levels of well-being.

Table 7 Per protocol analysis: Primary and secondary outcome measures at 6 months among randomized controls and those patients who highly adhered to the exercise program, and treatment effects (mean difference between the groups, adjusted for baseline values)

	Exercise			Control			6-month adjusted treatment	P-value
	Baseline	6-month	N	Baseline	6-month	N	difference (95% CI)	
Fatigue	40.7 (7.6)	42.6 (6.7)	35	43.5 (8.1)	43.7 (8.4)	35	0.7 (-2.0, 3.4)	0.60
FACT-Functional	20.2 (6.3)	21.2 (5.0)	35	22.6 (4.2)	21.3 (6.0)	35	0.56 (-2.0, 3.2)	0.65
FACT-Emotional	20.3 (2.8)	20.3 (3.0)	35	19.9 (3.3)	20.1 (3.5)	35	-0.1 (-1.3, 1.1)	0.91
HADS Anxiety	4.6 (3.0)	4.8 (3.2)	35	5.2 (3.0)	4.6 (2.9)	33	0.8 (-0.3, 1.8)	0.15
HADS Depression	3.4 (2.5)	2.6 (2.2)	32	2.9 (2.4)	2.7 (2.5)	35	-0.3 (-1.3, 0.6)	0.49
% Fat	29.2 (8.0)	29.3 (7.6)	32	30.0 (10.7)	29.7 (10.8)	34	0.4 (-0.7, 1.4)	0.50
Muscle mass (kg)	50.2 (9.7)	50.5 (9.5)	33	53.1 (10.8)	53.4 (10.6)	34	0.0 (-0.8, 0.8)	0.95
Weight (kg)	75.4 (14.7)	75.8 (14.2)	30	81.5 (19.0)	81.5 (18.7)	34	0.2 (-1.0, 1.4)	0.73
Total PA (mean counts per minute)	309.4 (93.9)	302.8 (80.9)	30	352.8 (89.2)	342.3 (103.9)	29	7.0 (-40.9, 26.9)	0.68
Leg muscle strength (kg)	43.6 (25.6)	65.5 (23.9)	32	58.6 (20.6)	63.6 (23.1)	31	11.3 (1.3, 21.3)	0.03
Grip strength (kg)	28.1 (9.9)	29.8 (10.0)	30	33.2 (9.51)	34.0 (9.0)	34	0.4 (-1.4, 2.2)	0.65
VO_{2peak} (ml/kg/min)	17.9 (6.9)	19.1 (6.1)	35	20.7 (7.39)	20.0 (7.9)	31	1.3 (-1.0, 3.7)	0.26
Systolic BP (mmHg)	137.9 (19.6)	136.9 (24.4)	33	139.3 (18.12)	133.0 (17.4)	33	5.1 (-2.2, 12.4)	0.17
Diastolic BP (mmHg)	82.9 (12.1)	81.0 (13.0)	33	85.2 (11.77)	79.3 (12.1)	33	3.7 (0.1, 7.3)	0.04
C-RP (mg/L)	3.6 (4.4)	3.56 (5.1)	31	4.1 (4.43)	6.2 (11.3)	34	-2.2 (-6.2, 1.8)	0.27
IgA (g/L)	1.6 (1.2)	1.5 (1.2)	33	1.5 (1.11)	1.8 (1.9)	34	-0.3 (0.6, 0.0)	0.08
IgG (g/L)	11.7 (5.0)	11.7 (4.4)	33	11.4 (5.19)	11.9 (6.6)	34	-0.4 (-1.7, 0.9)	0.53
IgM (g/L)	0.5 (0.4)	0.6 (0.4)	33	0.7 (0.45)	0.7 (0.6)	34	0.0 (-0.1, 0.1)	0.88
Hemoglobin (g/L)	126.2 (15.8)	131.8 (13.5)	32	134.3 (11.53)	132.8 (12.2)	35	-0.1 (-3.5, 3.3)	0.95

FACT: Functional Assessment of Cancer Therapy, HADS: Hospital Anxiety and Depression Scale, PA: Physical activity, CI: Confidence interval. Higher scores of fatigue indicate lower fatigue levels. Higher scores in the functional, emotional, anxiety, and depression scales indicate higher levels of well-being.

Table 8 Mean ± SD of fatigue, leg strength, and VO_{2peak} of participants with clinical fatigue at baseline. The exercise group comprises of those with high adherence to the exercise program.

	Exercise	N	Control	N
Fatigue (FACIT-F score)				
Baseline	27.7 ± 3.6	7	25.0 ± 7.8	10
3-month	38.4 ± 5.1	7	28.1 ± 10.8	8
6-month	37.2 ± 5.2	6	34.7 ± 7.6	6
12-month	34.8 ± 6.1	5	28.5 ± 5.7	6
Leg strength (kg)				
Baseline	37.9 ± 16.2	7	56.7 ± 33.0	9
3-month	60.4 ± 28.3	7	60.2 ± 17.9	7
6-month	59.8 ± 17.8	6	67.0 ± 27.1	5
12-month	89.2 ± 17.0	3	63.0 ± 35.6	5
VO_{2peak} (ml/kg/min)				
Baseline	17.0 ± 4.4	7	16.4 ± 5.3	10
3-month	20.5 ± 5.9	7	16.6 ± 4.4	8
6-month	18.5 ± 4.9	6	15.8 ± 6.3	5
12-month	20.4 ± 5.1	5	17.6 ± 2.9	4

Table 9 Mean (SDs) of blood pressure (BP) and biomarkers and unstandardized coefficients of arm allocation for the modified intention to treat analysis with those who accepted the exercise intervention vs control at 3 and 6 months

	Exercise			Control			3-month adjusted treatment difference (95% CI)	P-value
	**Baseline**	**3-month**	**N**	**Baseline**	**3-month**	**N**		
Diastolic BP (mmHg)	83.1 (11.4)	79.7 (12.0)	44	84.2 (11.6)	81.8 (10.9)	36	-2.1 (-5.4, 1.2)	0.21
Systolic BP (mmHg)	134.6 (18.1)	134.4 (19.1)	44	138.2 (18.4)	137.1 (20.1)	36	-1.3 (-6.2, 3.7)	0.62
C-reactive protein	4.2 (5.4)	3.0 (4.0)	44	3.6 (4.1)	3.4 (4.2)	38	-0.4 (-2.0, 1.2)	0.60
IgA	1.8 (4.1)	1.3 (1.1)	45	1.5 (1.0)	1.4 (0.9)	38	-0.1 (-0.2, 0.1)	0.23
IgG	11.3 (5.8)	11.8 (6.1)	45	11.5 (5.0)	11.9 (5.7)	38	-0.1 (-1.2, 1.0)	0.84
IgM	0.5 (0.4)	0.5 (0.3)	45	0.7 (0.4)	0.7 (0.5)	38	0.0 (-0.1, 0.1)	0.73
Hemoglobin	127.0 (15.1)	128.8 (15.8)	44	132.2 (13.1)	133.7 (12.6)	38	-0.6 (-4.0, 2.8)	0.73
	**Baseline**	**6-month**		**Baseline**	**6-month**	**N**	6-month adjusted treatment difference (95% CI)	P-value
Diastolic BP (mmHg)	83.2 (11.8)	81.1 (12.6)	35	85.2 (11.8)	79.3 (11.9)	33	3.6 (0.1, 7.1)	0.047
Systolic BP (mmHg)	137.7 (19.1)	136.9 (23.7)	35	139.3 (18.1)	133.2 (17.2)	33	5.1 (-2.0, 12.3)	0.15
C-reactive protein	3.4 (4.3)	3.5 (4.9)	33	4.1 (4.4)	6.2 (11.3)	34	-2.2 (-6.1, 1.7)	0.26
IgA	1.5 (1.2)	1.5 (1.2)	35	1.5 (1.1)	1.8 (1.9)	34	-0.3 (-0.6, 0.0)	0.08
IgG	12.3 (6.1)	12.4 (5.8)	35	11.4 (5.2)	11.9 (6.6)	34	-0.4 (-1.7, 0.9)	0.54
IgM	0.6 (0.4)	0.6 (0.4)	35	0.7 (0.5)	0.7 (0.6)	34	0.0 (-0.1, 0.1)	0.94
Hemoglobin	128.4 (15.9)	131.8 (13.5)	34	134.3 (11.5)	132.8 (12.2)	33	3.65 (0.0, 7.1)	0.05

Figure 1 Mean leg muscle strength (kg) by treatment group and time point.

Measurements in the control group increase from baseline to 3 months, and then remain similar at 6 months. Measurements in the intervention group are generally lower than in the control group at baseline. Measurements in the intervention group increase from baseline to 3 months, sharper and higher than in the control group, and then increase a bit more from 3 months to 6 months, i.e. the treatment effect is large when looking at 3 months

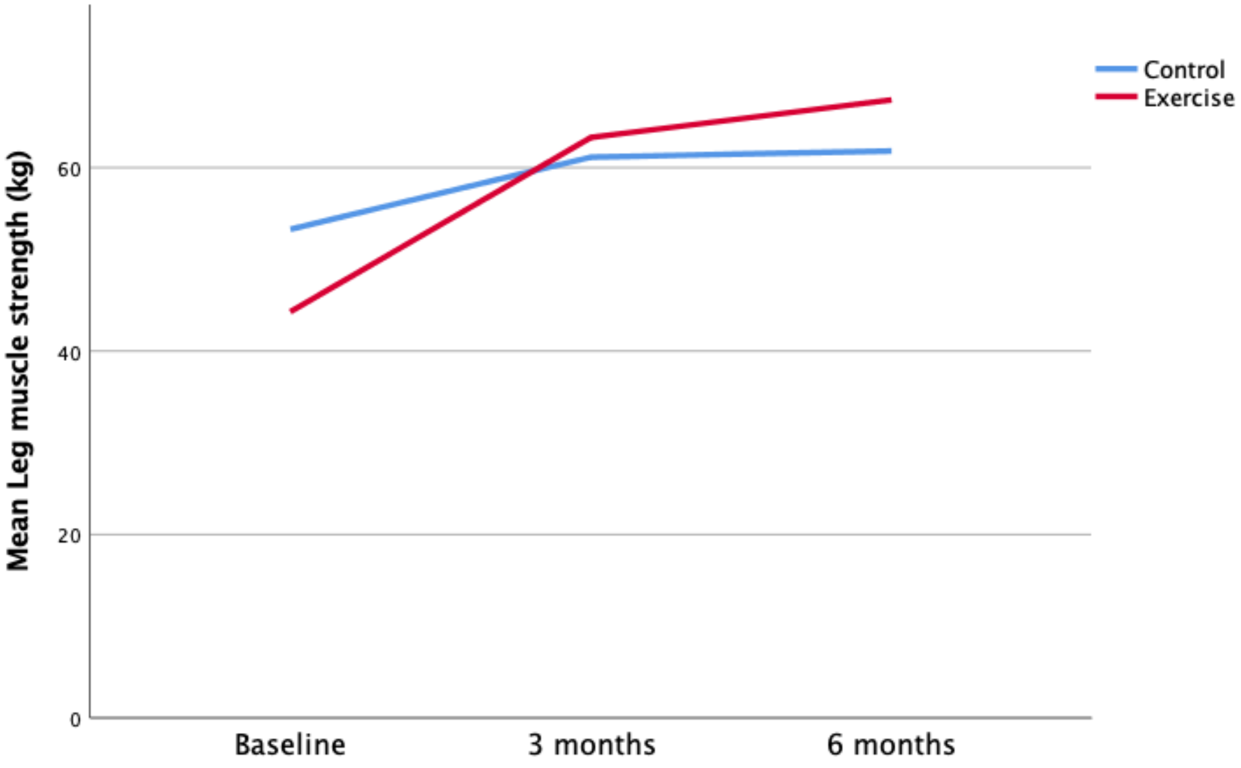

(or 6 months) compared to baseline, but small when looking at 6 months compared to 3 months.

Figure 2 Tukey plots for quality of life, anthropometry, and grip strength for all participants analyzed per protocol and those with clinical fatigue at baseline (FACIT-F score<3

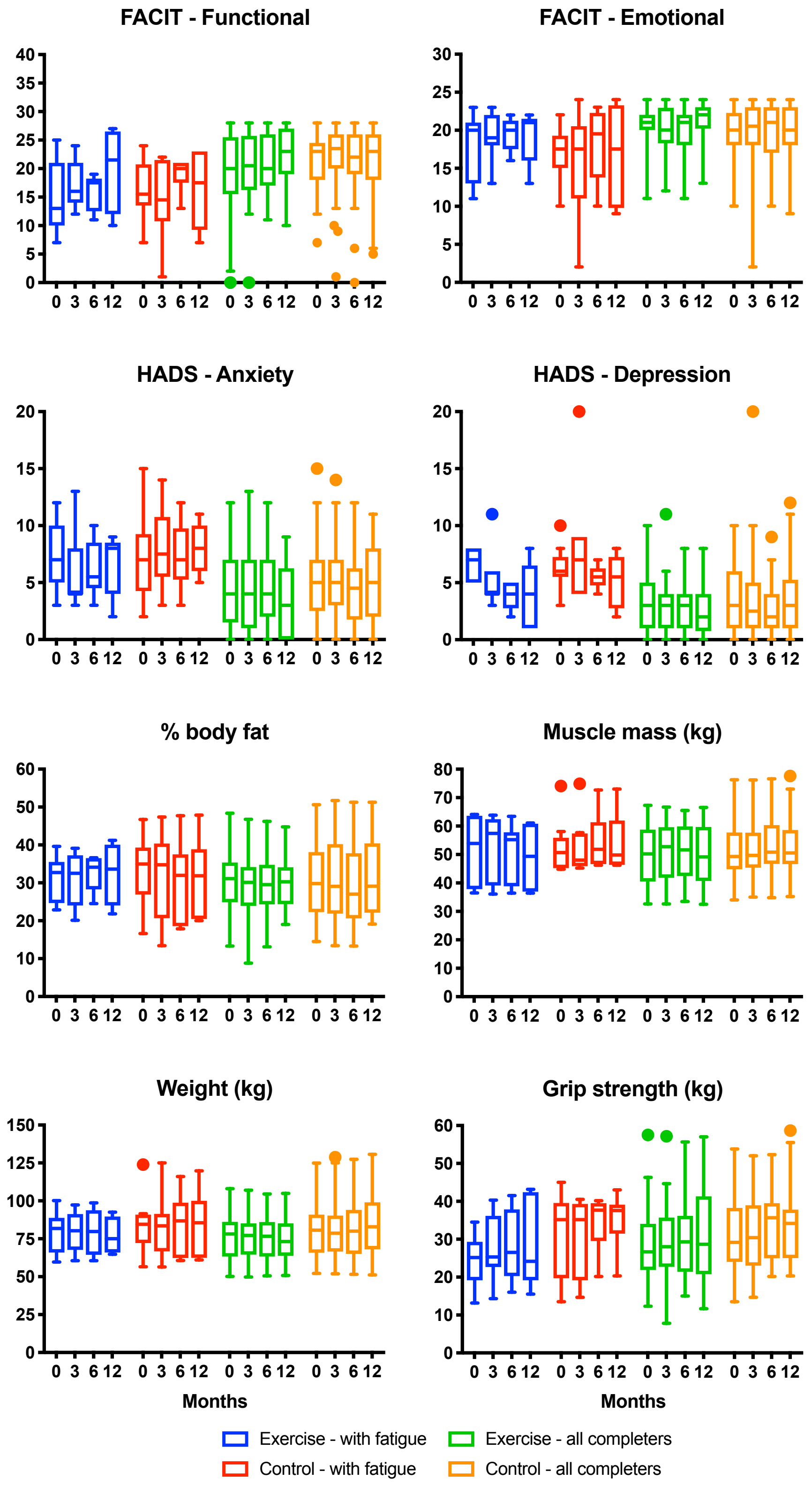

Study Protocol: Lifestyle study of patients with Multiple Myeloma

Chief Investigator:

Professor Kwee Yong

Professor of Haematology & Myeloma Lead Clinician

UCL Cancer Institute

72 Huntley Street
London WC1E 6BT

kwee.yong@ucl.ac.uk

Co-investigators:

Professor Jane Wardle

Director, Health Behaviour Research Centre

University College London

j.wardle@ucl.ac.uk

Bruce Paton

Clinical Specialist Physiotherapist

Institute of Sport, Exercise & Health

bruce.paton@uclh.nhs.uk

Dr Abigail Fisher

Senior Research Associate

University College London

abigail.fisher@ucl.ac.uk

Dr Rebecca Beeken

Research Psychologist

University College London

r.beeken@ucl.ac.uk

Aviva Cerner

Myeloma Clinical Nurse Specialist

Cancer Division, Haematology

aviva.cerner@uclh.nhs.uk

Professor Alan Hackshaw

Professor of Medical Statistics

CR:UK and UCL Cancer Trials Centre

a.hackshaw@ucl.ac.uk

Diane Gowers

Myeloma Research Nurse

Cancer Division, Haematology

Diane.gowers@uclh.nhs.uk

Rachael Hunter

Health Economist

University College London

r.hunter@ucl.ac.uk

Professor Fares Haddad

Director

Shirley D'Sa

Consultant Haematologist

Institute of Sport, Exercise & Health

fares.haddad@uclh.nhs.uk

Cancer Division, Haematology

Shirley.dsa@uclh.nhs.uk

Professor Andy Jewell

Kingston University and St Georges University of London

A.Jewell@sgul.kingston.ac.uk

Dr Ali Rismani

Consultant Haematologist

Whittington Hospital

a.rismani@nhs.net

Sponsor

University College London Hospitals NHS Foundation Trust

List of Abbreviations & Definitions

CNS: clinical nurse specialist

ECG: electrocardiogram

ECOG: Eastern Cooperative Oncology Group

EI: exercise intervention

HDT: high dose therapy

MRI: magnetic resonance imaging

CRF: cancer related fatigue

QoL: quality of life

Contents

Summary of study **19**

1.0 Background and Rationale **21**
Fatigue and health-related quality of life........ 21
Bone health 21

2.0 Study objectives and Design **22**
Primary objective: to test the hypothesis that a individually-tailored exercise programme in myeloma patients will improve symptoms of cancer related fatigue by clinically significant levels when compared to usual care **22**
Design **22**

3.0 Study outcome measures **22**
Primary outcome measure: **22**
Secondary outcome measures: **22**
Economic outcome measures: **23**
Feasibility outcome measures: **23**

4.0 Subject selection and recruitment **23**
Inclusion criteria: **23**
Exclusion criteria: 24

5.0 Consent and randomisation **24**

6.0 Trial interventions **25**
6.1 The exercise programme (EP) **25**
Aerobic Exercise Training 25
Resistance Training 26
Behavioural support 26
6.2 Control group **26**
6.3 Usual care **26**
6.4. Contact between months 6 and 12 .... **26**

7.0 Study visits and assessment **26**
7.1 Clinical and disease assessments **26**
7.2 Questionnaires **27**
7.3 Accelerometery for activity and sleep assessments **28**
7.4 Physiological assessments **28**
Determination of body mass and body composition 28
Measurement of resting blood pressure 28
Measurement of muscle strength and endurance 28
Assessment of exercise capacity and cardiorespiratory fitness 28
7.5 Qualitative Interviews **29**

9.0 Statistical considerations **29**
9.1 Pilot data **29**
9.2 Sample size calculation **29**
9.3 Recruitment rate **30**
9.4 Statistical analyses **30**
Primary outcome measure: 30
Secondary outcome measures: 30
Economic Analysis 30

10.0 Adverse events and reporting **31**
10.1 Definitions **31**
Adverse Reactions 31
Serious Adverse Events 31
Suspected Unexpected Serious Adverse Reactions 31
10.2 Reporting Adverse Events or Reactions **31**
10.3 Operational Definition – Serious Adverse Events **32**
Events not classed as SAEs 32
Expected SAEs 32

Expected SAEs related to Multiple Myeloma: ... 32
Expected SAEs common to all treatments: ... 32
Recording and Reporting SAEs and SUSARs ... 33

11. Trial Management and oversight ... 33
11.1 Oversight Committees ... 34
11.2 Trial Management Group (TMG) ... 34
11.3 Independent Data Monitoring Committee (IDMC) ... 34

12.0 Timescale ... 34

Protocol appendix 1. Modified International Myeloma Working Group Uniform Criteria of Response and Progression ... 39

Protocol appendix 2. ECOG Criteria ... 42

Protocol appendix 3. Eligibility & radiological screening of patients ... 43

Protocol appendix 4. Details of clinical assessments ... 45

Protocol appendix 5. Exercise program ... 46

Summary of study

This randomised controlled trial, embedded within a cohort study, in patients with multiple myeloma will further examine whether a 6 month exercise programme, when compared to usual care, will result in a clinically significant improvement in symptoms of fatigue, alongside improvements in quality of life and measures of fitness. Our trial builds upon a small single arm study, in which exercise appeared to improve outcomes in these particular patients, but it is now important to have a concurrent randomised control group. We will also collect novel data on the effects on markers of bone health.

Patients who have had stable disease for at least 6 weeks will be recruited from the Myeloma Clinics at University College London Hospital (UCLH), and asked to participate in a cohort study. Potentially eligible patients will provide informed consent. These patients will undergo a clinical assessment and screening, which will include a general physical examination, resting electrocardiogram (ECG), laboratory investigations, skeletal survey and MRI of the spine (if appropriate), to document extent and sites of skeletal disease. All of the follow up assessments will be done as part of this cohort study.

Soon after agreeing to participate in the cohort study, patients will be randomised to continue to receive usual care, or usual care plus a tailored exercise programme, delivered by specialist physiotherapists. However, to avoid contamination between the intervention groups (which can significantly affect the results and conclusions of trials of these types of interventions), only those randomly allocated to the exercise group will be informed of this, and a second consent sought. These patients will be asked to exercise three times a week for 6 months. In the first 3 months, one exercise session each week will be in the outpatient UCLH gym, under supervision, whilst in the second 3 months, patients will only attend once a month for supervised sessions. All other sessions will be home-based. Study outcomes will be obtained from blood tests, clinical and anthropomorphic assessments, QOL questionnaires, physical activity and sleep monitoring using accelerometry and assessments of exercise capacity, muscle strength and cardiovascular fitness.

Study visits will be at baseline (T0), three (T1), six (T2) and twelve (T3) months. At each timepoint there will be two visits one week apart, the first for clinical and questionnaire assessments, and the second for physiological assessments and to obtain accelerometer data. The total study period for each patient will be twelve months. Participant flow through the trial is highlighted in **Figure 1**.

Our trial will be used to design a larger multicentre phase III study, which if positive, could lead to an exercise programme being included as part of standard of care for myeloma patients. Such patients have already received various medical interventions (e.g. drugs and radiotherapy), so they could be supportive of a different type of intervention, which they can do at home. This might also have cost benefits to the NHS.

Figure 1: Participant Flow Chart

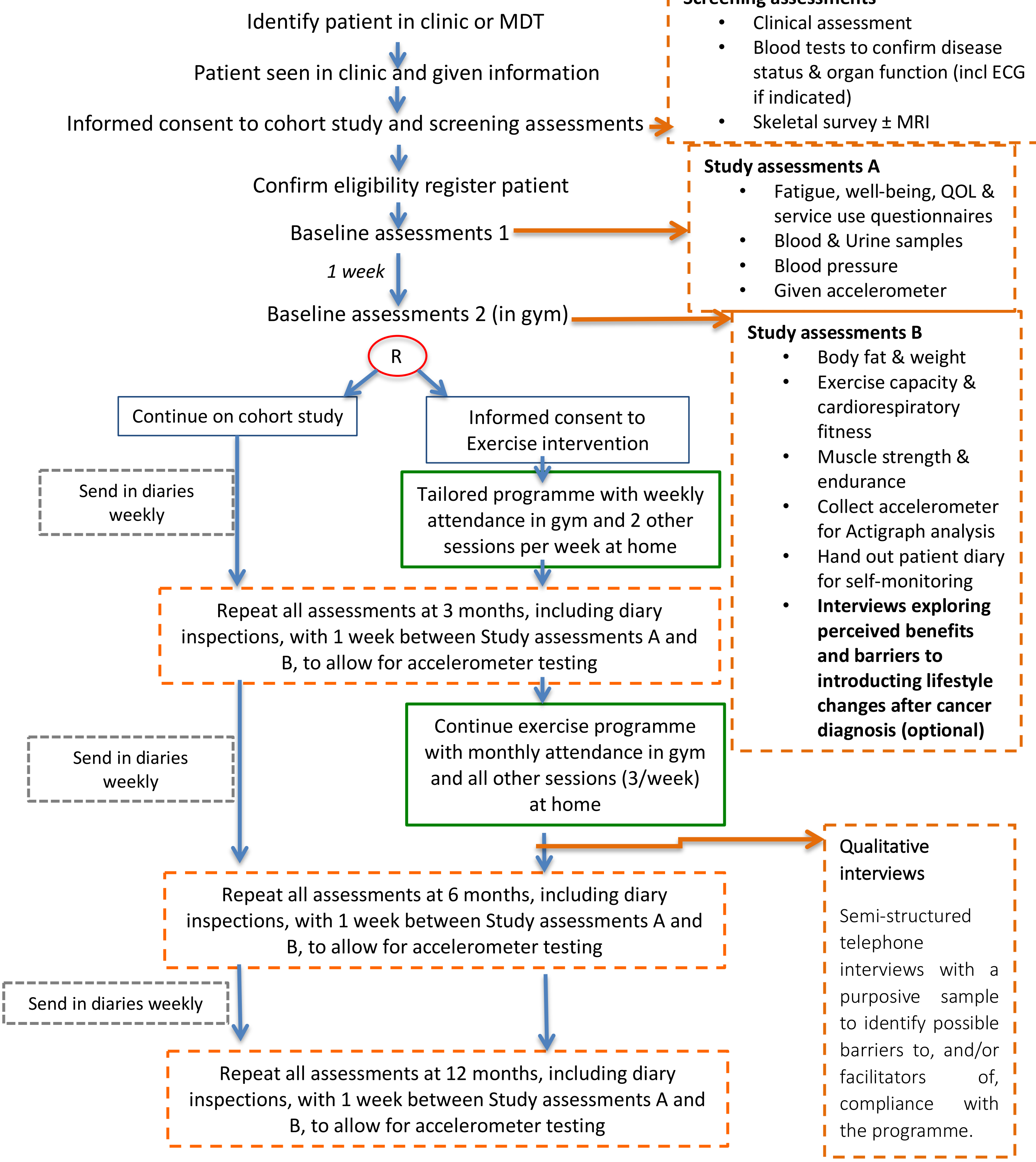

1.0 Background and Rationale

There is accumulating evidence that exercise is beneficial for cancer survivors (Schmitz et al., 2010). However, the majority of studies have focussed on breast, bowel and (recently) prostate cancer and there is need for research on other cancers (Bourke, Rosario, Copeland, & Taylor, 2012; Fong et al., 2012).

Multiple Myeloma (MM) is a plasma cell malignancy of the bone marrow that accounts for around 10% of all hematologic cancers, with approximately 4500 new cases each year in the UK (Kyle & Rajkumar, 2008). Clinical presentation is varied, but more common are symptoms of anaemia, bone pain, impaired renal function, hypercalcaemia and recurrent infections (Guiliani, Rizzoli, & Roodman, 2006). Of these, bone pain resulting from osteolytic bone disease, is the commonest symptom reported at diagnosis and may be related to skeletal complications such as fractures and vertebral collapse. Up to 80% of patients will suffer the effects of myeloma bone disease at some point during their myeloma journey (Guiliani et al., 2006). The majority of patients respond to chemotherapy, which is usually adminsitered in combination with high dose steroids, and enter a plateau phase when the disease is quiescent. This plateau phase generally lasts a median of 3 years with current therapies.

MM is incurable, but effective disease-directed therapies are extending the life expectancy of patients (Kumar et al, 2008), which substantially increases healthcare burden. Ther**efore efficacious** and cost-effective rehabilitation programmes are urgently required. MM survivors are a largely understudied group in this context, but are a patient population who suffer a number of serious and debilitating co-morbidities that have the potential to be improved by exercise.

Fatigue and health-related quality of life

Like many other cancer survivors, myeloma patients suffer a number of psychosocial problems, leading to anxiety and depression. Cancer-related fatigue is a clinical symptom that describes a lack of energy and tiredness that is not relieved by rest; it is reported in up to 70% of cancer patients during therapy or after surgery and can persist for many years after treatment (Lucia, Earnest, & Perez, 2003). Fatigue has multiple origins in cancer, including the side-effects of treatment, anaemia, sleep disturbances, reaction to tissue injury caused by disease, infections and psychosocial factors (Lucia et al., 2003). High levels of fatigue will likely reduce the physical activity levels of myeloma patients, which will in turn accelerate the physiological decline associated with the disease, and it can influence whether patients remain on maintenance treatment. This decline will be manifested as muscle wasting, weight changes, reduction in muscle strength and joint flexibility, and reduced fitness, thus reducing the ability to exercise or even perform everyday tasks and an overall decrease in quality of life (Burnham & Wilcox, 2002).

Two very recent Cochrane reviews concluded that exercise is beneficial for the management of cancer-related fatigue (Cramp & Daniel, 2010; Cramp & Byron-Daniel, 2012). Another reported that exercise interventions improved health related quality of life (Mishra et al., 2012). However, as with all areas of research in exercise and cancer, these findings were limited mainly to breast and prostate cancers (Cramp & Daniel, 2010).

Bone health

Patients with myeloma have abnormal bone metabolism, which is a direct result of their malignancy. The presence of myeloma cells in the bone marrow stimulates increased osteoclastic activity (bone absorption), that exceeds bone formation, thus resulting in net bone loss. This disruption of normal bone physiology occurs because of an imbalance in the receptor activator of NF-kappa-B-ligand (RANK-L)/osteoprotegerin (OPG) system (Giuliani et al, 2001). RANK-L, produced by T cells and osteoblasts, is a cytokine which acts on osteoclasts to stimulate bone resorption, while OPG acts as a natural decoy receptor, and inhibits the actions of RANK-L. In the bone marrow of patients with myeloma, production of RANK-L is increased, while that of OPG is reduced, leading to unopposed bone destruction. Raised serum levels of RANK-L predict a poorer prognosis (Terpos*, et al* 2004). In addition to lytic lesions, reduced bone mineral density and osteoporosis are often present in an accelerated form in myeloma patients. Bone morbidity is hence a major feature of the disease and contributes markedly to mortality and morbidity in these patients. Almost 80% of patients will have radiological evidence of skeletal involvement on the skeletal survey, most commonly affecting the vertebrae, ribs, shoulder, pelvis and long bones (Collins, 1998). Myeloma bone disease can cause severe bone pain and pathological fractures. Skeletal complications are difficult to manage, and patients frequently undergo surgical intervention to relieve cord compression, stabilise

the spine or as a pre-emptive measure, for example internal fixation to prevent fracturing a long bone. These in turn contribute to mortality and bone morbidity, with frequent admissions to hospital, the attendant infection risks and the ever-increasing need for complex pain relief. Although bisphosphonates have been proven to be beneficial to myeloma patients in large meta-analyses, their ability to prevent skeletal complications in individuals is limited. Thus there is an urgent need to explore other ways of impacting on bone disease. Serum and urinary markers of bone resorption and formation reflect activity of myeloma bone disease, but may also serve as useful indicators of bone dynamics in such patients (Terpos et al, 2010). No information is available on how such biochemical markers of bone metabolism may be affected by exercise.

There is a long established link between exercise and bone health in normal populations (MacDougall, et al 1992), with weight-bearing exercise in particular promoting bone mineral density and offsetting osteoporosis. A similar trend has also been found in menopausal women with weight-bearing and resistance exercise shown to improve bone mineral density (Bonaiuti, *et al* 2002). To our knowledge there have been no studies looking specifically at the effects of exercise on bone mineral density or bone function in cancer patients. Many studies allude to the likely benefits of exercise on maintaining bone mineral density, but most have used a multifaceted approach to bone health, including mineral supplements, hormonal supplements or bisphosphonates (Bae and Stein 2004, Smith 2003).

Very few exercise interventions have included myeloma patients. One small study in 24 patients demonstrated that exercise was feasible in MM patients, however the study was not powered to detect differences in outcomes (Coleman et al., 2003a). Evidence from a promising single arm pilot study carried out by Kwee Yong and colleagues at UCLH demonstrated that an individually tailored 6-month exercise programme in MM had high compliance, and that participants experienced a clinically significant improvement in fatigue and quality of life (Groenveldt et al., 2013). We now propose to confirm these results in a randomised-controlled trial (embedded in a cohort study) that will also allow us to gather preliminary data on the effects of exercise on bone health. We will also obtain preliminary information on cost-effectiveness, and collect feasibility outcomes. This information is vital for the design of a phase III RCT.

2.0 Study objectives and Design

Primary objective: to test the hypothesis that a individually-tailored exercise programme in myeloma patients will improve symptoms of cancer related fatigue by clinically significant levels when compared to usual care.

Secondary objectives: to examine exercise effects over the trial period on quality of life, body mass, body compostion, fitness and health indicators and to explore whether any differences in fatigue seen at 3 months are sustained/increase over 6 and 12 months. We will also collect feasibility outcomes, information for a cost-effectiveness analysis, and preliminary data on the effects of exercise on bone health in order to design and power a future larger randomised trial on exercise and bone health in patients with multiple myeloma.

Design

100 patients will be recruited in total. **Figure 1** illustrated the proposed flow of patients through the study**.** The study is a randomised trial embedded within a cohort study (see section 5).

3.0 Study outcome measures

Primary outcome measure:

- Fatigue, assessed using the 13-item Fatigue Scale of the Functional Assessment of Chronic Illness Therapy tool (FACIT) (Webster, Cella, & Yost, 2003)

Secondary outcome measures:

- Well-being, to be assessed using the FACT-G of the FACIT measurement system and the Hospital Anxiety and Depression scale (HADS)

- Intensity and frequency of physical activity to be assessed subjectively using Godin Leisure-Time Exercise Questionnaire
- Diet , physical activity, alcohol consumption, smoking and patients' need for lifestyle advice to be assessed using Health and Lifestyle Questionnaire
- Sleep quality will be assessed using Pittsburgh Sleep Quality Index
- Self-efficacy in chronic illness will be asessed using Self-Efficacy for Managing Chronic Disease 6-item Scale and additional three questions from the Self-Efficacy to Perform Self-Management Behaviors - Exercise Regularly Scale developed for exercise Chronic Disease Self-Management study (Lorig K et al 1996)
- Body mass and body composition: body fat and weight
- Blood pressure
- Vitamin D
- Muscle strength and endurance
- Exercise capacity and cardiorespiratory fitness
- Physical activity and sleep assessed using Actigraph accelerometer readings
- Blood and urine markers of bone health :
    - Biochemical parameters of bone metabolism: Serum TRACP-5b, OPG, RANKL, bALP and osteocalcin, urinary NTX excretion
    - Serum cytokine levels

Economic outcome measures:

- Generic health related quality of life to be assessed using the EuroQol-5D (EQ-5D) and compared with scores on the FACIT to determine sensitivity.
- Intervention costs
- Data on health service use to be collected using an amended version of the CSRI (Beecham & Knapp, 2001)

Feasibility outcome measures:

- Weekly recruitment rates
- Loss to follow up
- Compliance rates (number of sessions attended & diary completion)
- Basic costs

See section 7 for further details.

4.0 Subject selection and recruitment

Inclusion criteria:

(i) stable disease for at least 6 weeks, off treatment or on maintenance or **consolidation** treatment (see **Appendix 1** for full definition of disease stability)

(ii) ability to give informed consent

(iii) a good performance status (ECOG 0-2); see **Appendix 2** (Oken et al 1982)

(iv) clinically able to carry out an exercise training program on a regular basis (assessed by initial screening)

Patients would also be asked whether they would be willing to consider future research studies, such as dietary or exercise programs, in order to minimize the number of patients in the intervention group who decline the trial at

the second consent stage (see section 5). Patients who say clearly that they would not consider participating in such studies would not be included in the current one.

Exclusion criteria:

(i) spinal instability (as assessed on radiology in multi-disciplinary team (MDT)) meetings

(ii) those who have recently (within 4 weeks) had spinal or other surgery for pathological fractures

(iii) an abnormal resting ECG, where clinically indicated unexplained by further cardiological work-up

(iv) at risk of pathological fracture (Mirel's score, see **Appendix 3**)

(v) already participating in an exercise program as part of a research study

(vi) unstable angina

(vii) musculoskeletal disease limiting mobility

(viii) cognitive impairment that impedes ability to complete questionnaires

Patients who fit the inclusion criteria will be approached in person in the Myeloma Clinics. They will receive a patient information sheet on the Cohort study, and if interested in participating, will sign a consent form and undergo initial screening (see **Appendix 3** for details of screening). If enrolled in the study, a GP letter will be sent out.

Patients who are enrolled will then undergo a randomization to an exercise intervention programme, or usual supportive care.

5.0 Consent and randomisation

Randomised trials of lifestyle or behavioural changes are often difficult to analyse and interpret reliably because of potential 'contamination' in the control group, i.e. where people not randomised to the intervention being examined take up the intervention because they have been alerted to it when the trial was initially described (by the research nurse, clinician or Patient Information Sheet). The effect of contamination is to dilute the treatment effect (i.e. make it appear smaller than it really is). A recent example of contamination comes from the large US trial of prostate cancer screening, where 44% of the control group (men randomised to not have regular Prostate Specific Antigen testing) actually requested it from their physician during the trial, and this contributed to the lack of effect on the primary outcome measure (Andriole et al 2009); compared to the equivalent European trial which had low contamination and which did show a significant mortality benefit (Shroder et al 2009).

To minimise contamination in our study, we employ a study design (double consent) that has been successfully used before in a randomised trial of a behavioural intervention (Campbell et al 2005). In our study, the same double consent method will be used, as follows: All patients will be provided initially with an information sheet and consent form inviting them to participate in a cohort study aimed at allowing us to understand the relationship between parts of their lifestyle, physical health, biochemical markers and cancer management, and how these change over time in myeloma patients (**See Participant Information Cohort v1.0** and **Participant Consent Cohort v1.0** documents). Once consented to the cohort study, patients will be randomised to either the intervention group or 'remain in cohort' group (i.e. the control group), but only those in the intervention group will be informed of this, and they will receive a second information sheet and consent form, specifically asking them if they wish to also be considered for the exercise programme (**See Participant Information ETP v1.0** and **Participant Consent ETP v1.0**). However, the description of and consent to all methods of assessment and data collection are contained in the initial consent (i.e. for the cohort study), with the exception of the exercise log books that will be used to evaluate patient compliance to, and are therefore specific, to the exercise programme.

This approach is likely to be valuable where the intervention is viewed by patients as particularly desirable, meaning randomisation to a control group may cause distress. This is particularly pertinent in myeloma patients who have an incurable disease, often with rapid decline, at time of disease relapse. The main limitation of this double consent approach is the potential for patients who decline the exercise programme giving rise to potential bias, because the 'equivalent' group could still be in the control arm of the trial. However, this should only be a

problem if many patients decline the exercise programme, and our experience so far is that this would not be the case. Also, we will gauge interest for such a programme at the first consent stage.

The positive results of the one-arm pilot were well publicised in the UCLH clinic and many patients are aware of this and may wish to be given the exercise programme. In reality, until the results of the proposed trial are available, it is unclear whether exercise *is* beneficial over and above usual care in this patient group. Therefore both intervention and control patients are providing extremely valuable data. It is important that the control group are available (as far as possible) for comparison.

6.0 Trial interventions

6.1 The exercise programme (EP)

The EP (Protocol Appendix 5) will follow American College of Sports Medicine (ACSM) exercise guidelines for cancer survivors (Schmitz et al., 2010) and will follow the protocol of the successful one arm pilot, with the addition of a manualised behavioural support element including behaviour change techniques. Each patient will be intially assessed individually by the study physiotherapist with a subjective and physical examination. They will be given a programme based on this assessment, cardiopulmonary fitness and exercise capacity. Patients will be inducted to the gym in this session, and will undertake exercise training three times a week for a total of 6 months. In the first 3 months, one of the weekly exercise sessions will be performed as a supervised group in the Oupatient gym at UCLH and the other two sessions will be undertaken at home. Each session will comprise both aerobic and resistance exercise training in an aim to improve both cardiorespiratory fitness and muscle function. Patients may however perform the aerobic and resistance elements of each session on separate occasions if they wish. In the second 3 months, patients will perform exercises at home, and only attend the Outpatient gym for group sessions once a month.

At the time of exercise prescription patients will be told not to undertake exercise training on days that they feel extreme fatigue. Their baseline level for each exercise will be prescibed based on their initial assessment after induction to the gym. In addition, they will be provided with an alternative 'low-load' exercise session to be performed when they feel incapable of completing their usual training. They will be instructed to exercise as long as they do not feel pain, and this will also be monitored during the supervised sessions. Other musculoskeletal problems will be noted during the assessment and exercise prescription will take these problems into account.

Aerobic Exercise Training

Aerobic training will be in the form of walking; however patients may also exercise on a cycle ergometer, cross-trainer or stepper if it is more appropriate and convenient for them. The aerobic exercise training will start at a level appropriate for each patient (eg with 10 min bouts at 50% of heart rate reserve-75% of maximum (HRR) [Courneya, et al 2003]). Heart rate targets for these levels of exercise will be taken from baseline cardiopulmonary exercise testing. Patients will be provided with heart rate monitors to maintain the prescribed heart rate and therefore control exercise intensity. Bikes will be set to resistance levels, correlating with the above intensities during the VO2 peak test. To support monitoring of correct exercise intensity at home, patients will be asked to report their rating of perceived exertion (RPE) using the Borg Scale (see Borg Scale v1.0). This scale describes levels of exertion. The patient will be given a scale to take home, instructed in its use, and advised to work to levels of exertion as determined under supervision.

Gradual progression in the exercise training will be achieved by alternately increasing exercise duration by 5 min and exercise intensity by 5% heart rate reserve (HRR) every 4 weeks. All exercise programmes will be prescribed on an individual basis for each patient to ensure suitability, safety, and to promote adherence to the programme. The exercise intensity and/or duration may therefore be modified in individual patients, in accordance with the individual's functional capacity and ability to carry out the exercise.

Resistance Training

A number of exercises will be prescribed to target all major muscle groups (depending on individual health and contraindications). Resistance training will be performed on either gymnasium weightlifting equipment, body weight, or using elastic stretch bands of various resistances. These bands will be supplied to patients at the start of the EP to allow convenient home-based resistance training. Gradual progression in resistance training will be prescribed if deemed appropriate at each exercise session with the study physiotherapist.

Exercises have been chosen based on larger muscle group and function in UL / LL and Trunk + practicality and ability to perform at home . They are to be delivered in line with principles of resistance training (Kraemer & Ratamess, 2004).

Behavioural support

To complement the exercise intervention and enhance likelihood of longterm maintenance, participants will receive behavioural support based on Habit Theory and incorporating behaviour change techniques such as goal setting and self monitoring. Repetition of behaviour in a consistent context increases the likelihood of it becoming 'automatic', requiring minimal willpower or effort to engage in. Formation of a regular exercise routine with specific times and days and environments should increase the automaticity of exercise, increasing the chance of long-term maintenance. Physiotherapists will be provided with a manual to help them support their participants using behaviour change techniques. Within the behavioural sessions physiotherapists will help patients begin to plan how they will exercise more independently between months 3 – 6 (for example, looking in to local facitilies, indentifying barriers to exercise and problem solving).

6.2 Control group

The control group will receive usual care. Patients in the control group will be given a simple log book to record any exercise they take and asked to store and bring these for collection at 3, 6 and 12 month visits.

6.3 Usual care

All patients in the study will receive disease monitoring and bisphosphonates according to local policy, and any other supportive or disease-directed therapy that is standard of care for patients with myeloma.

6.4. Contact between months 6 and 12

We will have monthly telephone contact with both intervention and control participants between months 6 and 12. The phone calls will be an opporunity to remind participants to return monthly log books during this period and discuss any issues that have arisen.

7.0 Study visits and assessment

All outcome measures will be assessed at baseline [T0], and repeated measurements will be carried out at 3 months [T1] at 6 months [T2] and 12 months [T3]. At each timepoint there will be two study visits, one week apart (as per **Figure 1**). The first will involve the clinical assessment, questionnaires and delivery of accelerometer and the second the physiological assessments and collection of accelometer. The team member taking the assessments will be someone other than the team member delivering the intervention so that they are blinded to the group allocation.

7.1 Clinical and disease assessments

Standard clinical blood sampling procedures will be used. The biological measures to be obtained are highlighted in **Table 1**. These are further detailed in **Appendix 4.**

Table 1. Summary of biological measures to be obtained

Clinical screening outcome	Biological measures obtained	Purpose
Haematology & biochemistry	Full blood count, renal and liver function tests	Screening of suitability for EP
Immunology	Lymphocyte count, B and T cell numbers, T cell subsets, immunoglobin, NK cell numbers and function	Screening of suitability for EP / examine EP effects on immune function
Biochemical parameters of bone metabolism	Serum TRACP-5b, OPG, RANKL, bALP and osteocalcin, urinary NTX excretion.	Examine EP effects on bone health
Serum cytokine levels	IL-6, TNF, CRP	Examine EP effects on markers of fatigue and inflammation

7.2 Questionnaires

Fatigue will be measured using the 13 item Fatigue Scale of the Functional Assessment of Chronic Illness Therapy (FACIT) (Webster, Cella, & Yost, 2003). The FACIT Measurement System is considered appropriate for use with patients with any form of cancer and importantly has been shown to be responsive to change in clinical and observational studies (Webster et al., 2003). Quality of life will be assessed using the FACT-G of the FACIT measurement system (Webster et al., 2003). This measure has shown positive response to exercise in other cancers (Mishra et al., 2012). Patients will also complete the Hospital Anxiety and Depression scale (HADS) a widely used psychometric measure (Zigmond & Snaith, 1983).

For our cost-effectiveness analyses we will ask patients to complete the EuroQol-5D (EQ-5D,), a 5 item, 3 level questionnaire, where the resulting 243 health states have associated preference scores obtained from the general population. The EQ-5D is the generic health related quality of life measure that is prescribed by NICE for use in economic evaluations for the calculation of Quality Adjusted Life Years (QALYs). The evidence regarding the responsiveness of the EQ-5D to changes in myeloma or fatigue is limited and contradictory. Some studies have found the EQ-5D to be responsive in this patient group (Kvam et al 2011) and to capture changes in fatigue (Strickland et al 2012), whereas reviews have reported evidence of poor responsiveness (Brazier et al 2007, NICE DSU 2010). The ED-5D will therefore be validated against the disease-specific measure of QOL, the FACT-G, and scores will also be compared with our measure of fatigue. This will inform our decision about how to measure quality of life in a main trial. Patients will also complete an amended version of the CSRI (Beecham & Knapp, 2001) to capture participants resource use. **As part of this questionniare we will also ask patients to indicate** if their use of health/social care services was related to myeloma or another, health condition unassociated with their cancer diagnosis. This will enable us to assess the impact of the intervention on the healthcare costs associated with treatment and management of myeloma more acurately.

In addition patients will be asked to complete a Health and Lifestyle questionniare which will inlcude questions about their diet and other health behaviours such as smoking and alcohol consumption. The questionniare will also assess patients' needs for additional lifestyle advice.

We will also explore the patients quality of sleep which will be assessed using Pitsburg Sleep Quality Index questionniare. We will also be interested in exploring the frequency and the level of intensity of physical activity the participants may be engaging in. This will be asessed using Godin Leisure-Time Exercise Questionnaire. We will also be measuring patients' perception of their own confidence in their own ability to carry out behaviours

such as physical activity. This will be measured by using the adapted version of 'Self-efficacy in chronic conditions' scale.

All **9** questionnaires will be completed by patients in clinic with the assistance of a researcher. Examples of these questionnaires are provided.

7.3 Accelerometery for activity and sleep assessments

Physical activity and time spent in light, moderate and vigorous physical activity will be measured objectively using the Actigraph (Ambulatory Monitoring, Ardsley, NY) following the protocol used in a previous feasibility study in myeloma. The Actigraph is the most commonly used accelerometer in adult and field based physical activity research. It is a device similar in size and shape to watch worn on the **waist or** wrist, which senses and stores physical motion. The Actigraph signals are processed online, and stored data are transferred to a computer for display, interpretation, and conversion into activity parameter. The Actigraph has been used in varied clinical populations and compares well against other devices for Validity reliability (van Remoortel et al., 2012) and against physical activity questionnaires. The participation in light, moderate and vigorous activity will also be assessed subjectively using mentioned earlier Godin Leisure-time exercise (Godin and Shephard, 1990).

Sleep will also be measured with the Actigraph. On healthy people it has a minute-by-minute agreement of 85% to 95% between activity-based sleep-wake scoring and traditional polysomnography-based scoring. A number of aspects of sleep from sleep onset latency, quality and length can be obtained from the Actigraph. Patients recorded their bedtimes and rise times on a log during the **7** day measurement periods. This follows the measurement procedure used by Coleman et al in their Multiple myeloma feasibility study (Coleman et al., 2003b; Sadeh, Sharkey, & Carskadon, 1994). **Patients will also be required to complete the Pittsburgh Sleep Quality Index (PSQI, Buysse et al, 1989).**

7.4 Physiological assessments

Each patient will perform the physiological assessment at approximately the same time of day on each testing occasion.

Determination of body mass and body composition

Body mass and composition will be measured at each testing session. Body fat will be assessed via bioelectrical impedance analysis (BIA). This is a cost-effective and non-invasive method used to estimate total body fat. It has a high level of precision when compared to other more invasive indirect methods of body fat determination.

Measurement of resting blood pressure

Resting blood pressure will be measured via an electronic sphygmomanometer before patients perform any of the exercise tests.

Measurement of muscle strength and endurance

Isometric muscle strength (hand grip strength) will be determined using hand-held dynamometry. This is a common method that is used extensively to assess general strength characteristics. The lower limb strength will be measured using a leg press, a linear encoder and relevant computer software (MuscleLab, Ergotest, Norway). An estimated 1RM and peak power value will be determined from force-velocity data obtained by asking the patient to perform a number of sets of lifts at different submaximal loads (Bosquet et al. 2010).

Assessment of exercise capacity and cardiorespiratory fitness

Participants pulmonary function and cardiopulmonary fitness will be determined using an incremental ramp protocol test with a bicycle ergometer and cardiopulmonary exercise testing (CPET) equipment (Cortex Metalyser 3B).. VO_{2max} is the gold standard measure of cardiorespiratory fitness and an individual's exercise capacity, however true $VO2_{max}$ is difficult to ellicit in this population group due to symptoms that may limit exercise

capacity such as fatigue or muscle weakness. For this reason the values of $VO2_{peak}$ and anaerobic threshold will be used to express participants' cardiopulmonary fitness (Steins Bisschop et al, 2012).

7.5 Qualitative Interviews

Patients who will agree to take part in the cohort study will also be offered the opportunity to give an interview, during which they would discuss their experience of receiving lifestyle advice after Multiple Myeloma diagnosis, their beliefs about the importance and timing of the delivery of such advice and their views about faciltators and barriers to health-related lifestyle change after cancer diagnosis. The patients will be encouraged to share their thoughts on how feasible it is to introduce such lifestyle changes and will be asked to comment on their own experience of seeking lifestyle advice, introducting lifestyle changes into their own lives or why they think such changes may or may not be feasible to adopt within their current lifestyle. This interview will provide an in-depth account of patients' experience of seeking and receiving lifestyle advice and practical issues related to adopting health advice after cancer diagnosis. This will help us to better understand patients' needs for lifestyle advice and identify obstacles and factors conductive to making healthful lifestyle choices. Patients will be informed that taking part in the interviews will be optional. The interview will take place after all other assessments are completed, at the end of the second assessment day. The interview schedule is enclosed with the application.

We will conduct semi-structured individual telephone interviews with a purposive sample from the intervention group (n ~ 20) who are between weeks 8 and 10 of the exercise programme. The interviews will allow us to explore, in a bit more depth, patient experiences of the trial and exercise programme and any self- reported barriers to, and facilitators of, compliance with the programme.

All interviews will be audio recorded and transcribed. Thematic analysis will be used to identify the main themes that will form the basis of our results. Results from the interviews will inform the design of the larger trial and may lead to refinements of our intervention.

9.0 Statistical considerations

9.1 Pilot data

In the pilot study acceptance rates were high (80% of patients approached), and the attrition rate (24%, all prior to start of program, due to disease progression, screen failures or personal reasons) was similar to that reported for exercise programs in other cancer populations (Groeneveldt et al, 2013). All of the 37 patients who started the exercise program completed 3 months of the study, and attendance and adherence rates were high (86% and 97% respectively). Funding constraints meant that only 28 patients completed 6 months. Significant improvements in CRF (assessed by fatigue subscale of the FACIT measurement system), quality of life (QOL; Functional Assessment of Cancer Therapy General Scale (FACT-G)) and limb strength were observed. In the 28 patients who completed 6 months, CRF (assessed by FACIT subscale of the measurement system) scores improved by +4.4 ($p=0.0006$) and mean QOL total scores improved by +7.3 at 6 months ($p<0.001$). Upper and lower limb strength also improved significantly ($p<0.001$ and $p<0.01$ respectively) by 3 months, and this effect was maintained at 6 months. Patients reported very high satisfaction with the program, with some individuals reporting dramatic changes in lifestyle.

9.2 Sample size calculation

This trial is powered to detect a clinically significant (Cella, Eton, Lai, Peterman, & Merkel, 2002) 4 point change in the mean fatigue scores between the intervention and control groups; an effect size of 0.69 (using a standard deviation of 5.8 from our pilot data). This requires 68 patients in total (80% power, 2-sided 5% statistical significance), but the target is to recruit 100 participants to allow for approximately a third drop-out during the exercise program, as observed in the pilot study. However, in the process of recruitment we have learned that about half of patients who are allocated to the intervention decline. Therefore, we need n=52 to start the exercise program (to yield 34 who complete), so we need to allocate n=104 (to get n=52 to start the program). Hence, the randomization allocation ratio 3:1 (104:34; intervention: control) and new recruitment target of 138 patients. Changes from baseline will be assessed for all outcome measures**.** Based on our pilot data it is expected that changes in QoL, functional well-being and physiological function will be apparent during the first 3 months.

9.3 Recruitment rate

Recrutiment is planned to take place over 24 months. Patients will be recruited from the specialist myeloma clinics at UCLH and Barts. Approximately 150-200 patients are seen annually in each centre who are in plateau phase, either off treatment or on maintenance therapy. We expect that approximately a third of these will consent to the cohort study, of whom 70% will pass screening. The flow of patients through the study may also depend upon availability of gym facilities for the programme, and the availability and training of physiotherapists or sports physiologists.

9.4 Statistical analyses

Primary outcome measure:

Fatigue will be analysed using repeated measures (mixed modelling), over the 3 time points (3, 6 and 12 months), after allowing for the baseline measure of fatigue. There will also be focus on the effect at 3 months, analysed by linear regression. Both intention to treat and per protocol analyses will be carried out.

Secondary outcome measures:

Patients in the exercise programme who drop out during the trial will be summarised as a frequency table, showing the proportion of patients who start the programme who are still exercising at each of the 3 time points (3, 6 and 12 months later).

Quality of life (FACT-G of the FACIT), and Hospital Anxiety and Depression scale (HADS), will be converted into their standard scores and domains and analysed using linear regression (for the effect by 3 months) and mixed modelling/repeated measures (for all time points). The other outcome measures (physical and exercise capacity endpoints) will also be analysed using the same methods, as will the biochemical markers.

Assessments for each outcome will be made to determine whether the data are Normally distributed. For outcomes that are not, even after appropriate transformations, non-parametric methods will be used for data analyses at specific time points.

Missing data will be dealt with using methods such as those summarised in http://missingdata.lshtm.ac.uk/talks/RSS_2012_04_18_James_Carpenter.pdf, or chained equations (Royston & White 2001).

Economic Analysis

The EQ-5D will be validated against the the FACT-G, and scores will also be compared with our measure of fatigue, to give us an indication of the suitability of using the EQ-5D for the health economics analysis in a main trial. If the EQ-5D is found to be a suitable measure of QOL in this population, we will calculate the quality adjusted life years for each patient using the standard formula from Dolan (1997). If not, we will investigate other strategies such as mapping or calculating cost per change in another quality of life measure.

We will calculate the costs associated with implementing and delivering the exercise intervention. Use of health services for the intervention and control group, will be collected from patient records and costed at national rates using reference cost data.

The planned cost effectiveness analysis is to examine the incremental cost per quality adjusted life year gained (QALY) (ICER) of the exercise intervention compared to usual care for the duration of the trial from the health care perspective. Due to the small sample size there is likely to be a lot of uncertainty associated with the results and the trial will not be powered to detect differences between the two groups. We will use non-parametric methods though for calculating confidence intervals around the ICER based on bootstrapped estimates of the mean cost and QALY differences. The bootstrap replications will also be used to construct a cost-effectiveness acceptability curve, which will show the probability that the exercise intervention is cost-effective compared to usual care for different values of the NHS' willingness to pay for an additional QALY gained. We will also subject the results to deterministic (one-, two- and multi-way) sensitivity analysis.

10.0 Adverse events and reporting

10.1 Definitions

An adverse event (AE) is any untoward medical occurrence in a participant or clinical trial participant which does not necessarily have a causal relationship with this treatment and can include;

- any unintentional, unfavorable clinical sign or symptom
- any new illness or disease or the deterioration of existing disease or illness
- any clinically relevant deterioration in any laboratory assessments or clinical tests.

Adverse Reactions

- Adverse reactions are all untoward and unintended responses to the intervention.
- All AEs judged by either the Investigator or the Sponsor as having a reasonable suspected causal relationship to the intervention qualify as adverse reactions. The expression "reasonable causal relationship" should convey that there are facts (evidence) or arguments to suggest a causal relationship

Serious Adverse Events

A Serious Adverse Event (SAE) is defined in general as 'any untoward medical occurrence or effect that:

1. results in death,
2. is life-threatening*,
3. requires inpatient hospitalization or prolongation of existing hospitalization,
4. results in persistent or significant disability or incapacity,
5. consists of a congenital anomaly or birth defect or
6. may jeopardize the participant and may require medical or surgical intervention to prevent one of the outcomes listed above.'

*The term life-threatening refers to an event in which the participant was at risk of death at the time of the event; it does not refer to an event which hypothetically might have caused death if it was more severe.

Medical judgement should be exercised in deciding whether an SAE is serious in other situations. Important AE/ARs that are not immediately life-threatening or do not result in death or hospitalization but may jeopardize the participant or may require intervention to prevent one or the other outcomes listed in the definition above, should also be considered serious.

Where an SAE is deemed to have been related to the intervention used within the trial the event is termed as a **Serious Adverse Reaction (SAR)**.

Suspected Unexpected Serious Adverse Reactions

A Suspected Unexpected Serious Adverse Reaction (SUSAR) is a Serious Adverse Reaction which also demonstrates the characteristics of being unexpected, the nature, seriousness, severity or outcome of which is not consistent with the information about the medicinal product in question set out in the Summary of Product Characteristics or Investigator Brochure.

10.2 Reporting Adverse Events or Reactions

Information about adverse events whether volunteered by the participant, discovered by investigator questioning or detected through physical examination, laboratory test or other investigation will be collected and recorded on

the relevant CRF.

Adverse reactions will be collected for all participants from the time of registration until 30 days after the date of last intervention and will be evaluated for duration and intensity according to the National Cancer Institute Common Terminology Criteria for Adverse Events V4.0 (NCI-CTCAE). A copy is provided in the Investigator Site File and may be obtained at: **http://evs.nci.nih.gov/ftp1/CTCAE/About.html**

CTCAE_4.03_2010-06-14_QuickReference_8.5x11.pdf

Published date: 28 May 2009.

10.3 Operational Definition – Serious Adverse Events

Events not classed as SAEs

The following events **will not** be recorded as SAEs within this trial:

Hospitalization for:

- Routine treatment or monitoring of the condition (myeloma) not associated with any deterioration in condition.
- Treatment which was elective or pre-planned, for a pre-existing condition not associated with any deterioration in condition.
- Admission to hospital or other institution for general care, not associated with any deterioration in condition.
- (Overnight) admission to hospital for administration of blood products, or for monitoring
- Treatment on an emergency, outpatient basis for an event not fulfilling any of the definitions for serious as given above and not resulting in hospital admission.
- Disease progression
- Deaths attributable to multiple myeloma beyond 30 days of the last administration of the study agent

Expected SAEs

The following events **will be** classed as expected SAEs within this trial and therefore will not be reportable as SUSARs unless the Investigator considers the severity to be unexpected:

Expected SAEs related to Multiple Myeloma:

- Hypercalcemia
- Pain control necessitating admission to hospital
- Infections requiring intravenous antibiotics
- Blood product support necessitating admission to hospital
- Spinal cord compression
- Renal failure
- Fractures and / or corrective surgery

Expected SAEs common to all treatments:

- Anemia
- Neutropenia
- Thrombocytopenia

- Infections requiring intravenous antibiotics
- Nausea/Vomiting
- Bowel disturbance
- Extravasation
- Hyperglycemia

Recording and Reporting SAEs and SUSARs

All SAEs / SUSARs occurring for all participants from the time of randomization until 30 days after the date of last intervention must be recorded on the SAE or SUSAR Form and faxed to the CTRU within 24 hours of the research staff becoming aware of the event. Once all resulting queries have been resolved, the CTRU will request the original form be posted to the CTRU and a copy to be retained on site:

For each SAE / SUSAR, the following information will be collected:

- full details in medical terms with a diagnosis, if possible
- CTCAE grade
- its duration (start and end dates if applicable)
- action taken
- outcome
- causality (i.e. relatedness to trial drug / investigation), in the opinion of the investigator) *
- whether or not the event would be considered expected or unexpected (see section 15.4) *

Additionally, any SAE occurring in a participant who has previously received a Celgene product (Thalidomide, Lenalidomide or Pomalidomide) prior to the start of the Study or is currently receiving a Celgene product during the study, should be assessed for causality to the Celgene product. The Investigator/Sponsor shall report to Celgene's Drug Safety Department **within 24 hours** of the Investigator's knowledge any **SAE considered related to the Celgene product (this will be reported as an SAR).**

*Assessment of causality and expectedness must be made by a doctor. If a doctor is unavailable, initial reports without causality and expectedness assessment should be submitted to CTRU within 24 hours but must be followed up by medical assessment as soon as possible thereafter.

Please ensure that each event is reported separately and not combined on one SAE form.

Any follow-up information should be faxed to the CTRU within 24 hours of the research team becoming aware of the information. Events will be followed up until the event has resolved or a final outcome has been reached.

11. Trial Management and oversight

Participating sites must agree to allow trial-related on-site monitoring and Sponsor audits by providing direct access to source data/documents as required. Patients are informed of this in the patient information sheet and are asked to consent to their medical notes being reviewed by appropriate individuals on the consent form.

The Health Behaviour Research Centre will be responsible for the day to day coordination and management of the trial and will act as custodian of the data generated in the trial (on behalf of UCL). The centre is responsible for all duties relating to safety monitoring.

11.1 Oversight Committees

11.2 Trial Management Group (TMG)

The TMG will include the Chief Investigator, clinicians and experts from relevant specialities (see pages 1-2_. The TMG will be responsible for overseeing the trial and will meet regularly (for example every 6 months).

The TMG will review substantial amendments to the protocol prior to submission to the REC. All investigators will be kept informed of substantial amendments through their nominated responsible individuals.

11.3 Independent Data Monitoring Committee (IDMC)

The role of the IDMC is to provide independent advice on data and safety aspects of the trial. Meetings of the Committee will be held once per year to review interim analyses, or as necessary to address any issues. All members will be required to sign the IDMC charter.

12.0 Timescale

Set up: June-August 2013

Recruitment: August 2013-August 2015

Baseline assessments: August 2013-September 2015

3 month follow up assessments: November 2013-January 2015

6 month follow up assessments: February 2014-April 2016

12 month follow up assessments: August 2014-October 2016

Analysis and interpretation of results: October 2016-December 2016

Total duration: 3 years, 6 months

13.0 References

Andriole, G.L. et al. (2009) Mortality results from a randomised prostate cancer screening trial, NEJM, 360, 1310-19.

Bae, D.C. & Stein B.S. (2004). The diagnosis and treatment of osteoporosis in men on androgen deprivation therapy for advanced carcinoma of the prostate. The Journal of Neurology, 172, 2137-44.

Baechle, T.R. (editor). (1994). Essentials of Strength Training and conditioning. National Strength and Conditioning Association. 259.

Beecham, J. and Knapp, M. Costing psychiatric interventions, in G.Thornicroft (ed.) Measuring Mental Health Needs, 2001, Gaskell, 2nd edition, 200-224.

Blade, J., et al. (1998) Criteria for evaluating disease response and progression in patients with multiple myeloma treated by high-dose therapy and haemopoietic stem cell transplantation. Myeloma Subcommittee of the EBMT. European Group for Blood and Marrow Transplant. Br J Haematol, 102, 1115-1123.

Borg, E. & Borg, G. 2002. A comparison of AME and CR100 for scaling perceived exertion. Acta psychologica, 109, (2) 157-175

Borg, E. & Kaijser, L. 2006. A comparison between three rating scales for perceived exertion and two different work tests. Scandinavian Journal of Medicine and Science in Sports, 16, (1) 57-69 available from: http://www.scopus.com/inward/record.url?eid=2-s2.0-33645101648&partnerID=40&md5=a97d958329702d876bb12770610558fe

Borg, G. & Dahlstrom, H. (1962). A case study of perceived exertion during a work test. Acta Societatis Medicorum Upsaliensis, 67, 91-93 available from: http://www.scopus.com/inward/record.url?eid=2-s2.0-47849122143&partnerID=40&md5=5dfa8aee043c274322ae34a1488580f6

Borg, G.A.V. 1982. Psychophysical bases of perceived exertion. Medicine and Science in Sports and Exercise, 14, (5) 377-381 available from: http://www.scopus.com/inward/record.url?eid=2-s2.0-0020410785&partnerID=40&md5=e4400a472c223331cbf97737d40e7780

Bosquet, L., Porta-Benache, J. & Blais, J (2010). Validity of a commericial linear encoder to estimate bench press 1 RM from the force-velocity relationship. Jounrnal of Sports Science and Medicine, 9, 459-463.

Bourke, L., Rosario, D., Copeland, R., & Taylor, S. (2012). Physical activity

for cancer survivors. BMJ, 344, d7998.

Buysse DJ, Reynolds CF, Monk TH, Berman SR, Kupfer DJ (1989). The Pittsburgh Sleep Quality Index: Anew instrument for psychiatric practice and research. Psychiatry Research, 28,193-213.

Brazier, J., Yang, Y. and Tsuchiya, A. (2007). Review of methods for mapping between condition specific measures onto generic measures of health. Office of Health Economics. NICE Decision Support Unit (2010) The incorporation of health benefits in cost utility analysis using the eq-5d available from: http://www.nicedsu.org.uk/PDFs%20of%20reports/DSU%20EQ5D%20final%20report%20-%20submitted.pdf

Burnham, TR. & Wilcox, A. (2002). Effects of exercise on physiological and psychological variables in cancer survivors. Medicine & Science in Sport and Exercise, 34 (12), 1863-7.

Campbell, R., Peters, T., Grant, C., Quilty, B., & Dieppe, P. (2005). Adapting the randomized consent (Zelen) design for trials of behaviour interventions for chronic disease: feasibility study. J Health Service Research Policy, 10, 220-225.

Carson, C.P., Ackerman, L.V. & Maltby, J.D. (1955) Plasma cell myeloma; a

clinical, pathologic and roentgenologic review of 90 cases. Am J Clin Pathol, 25, 849-888.

Cella, D., Eton, D. T., Lai, J., Peterman, A. H., & Merkel, D. E. (2002). Combining anchor and distribution-based methods to derive minimal clinically important differences on the Functional Assessment of Cancer Therapy (FACT) Anemia and Fatigue Scales. Journal of Pain and Symptom Management, 24, 547-561.

Chen, M.J., Fan, X. & Moe, S.T. (2002) Criterion-related validity of the Borg

ratings of perceived exertion scale in healthy individuals: a meta-analysis. J Sports Sci, 20, 873-899.

Coleman, E.A., Coon, S., Hall-Barrow, J., Richards, K., Gaylor, D. & Stewart,

B. (2003a) Feasibility of exercise during treatment for multiple myeloma. Cancer Nurs, 26, 410-419.

Coleman, E. A., Coon, S., Hall-Barrow, J., Richards, K., Gaylor, D., & Stewart, B. (2003a). Feasibility of exercise during the treatment for multiple myeloma. Cancer Nursing, 26, 410-419.

Coleman, E.A., Hall-Barrow, J., Coon, S. & Stewart, C.B. (2003b) Facilitating exercise adherence for patients with multiple myeloma. Clin J Oncol Nurs, 7, 529-534, 540.

Collins, C. (1998). Multiple myeloma. In: Imaging in Oncology (ed. by J.E.Husband & R.H.Resnik), Vol 2.

Coon, S.K. & Coleman, E.A. (2004) Exercise decisions within the context of multiple myeloma, transplant, and fatigue. Cancer Nurs, 27, 108-118.

Courneya, K.S., Friedenreich, C.M., Sela, R.A., Quinney, H.A., Rhodes, R.E. & Jones, L.W. (2004) Exercise motivation and adherence in cancer survivors after participation in a randomized controlled trial: an attribution theory perspective. Int J Behav Med, 11, 8-17.

Courneya, K.S., Mackey, J.R., Bell, G.J., Jones, L.W., Field, C.J. & Fairey, A.S. (2003) Randomized controlled trial of exercise training in postmenopausal breast cancer survivors: cardiopulmonary and quality of life outcomes. J Clin Oncol, 21, 1660-1668.

Cramp, F. & Byron-Daniel, J. (2012). Exercise for the management of cancer-related fatigue in adults (Review). Cochrane Reviews, 1-95.

Dimeo, F., Bertz, H., Finke, J., Fetscher, S., Mertelsmann, R. & Keul, J. (1996) An aerobic exercise program for patients with haematological malignancies after bone marrow transplantation. Bone Marrow Transplant, 18, 1157-1160.

Dimeo, F.C., Tilmann, M.H., Bertz, H., Kanz, L., Mertelsmann, R. & Keul, J. (1997) Aerobic exercise in the rehabilitation of cancer patients after high dose chemotherapy and autologous peripheral stem cell transplantation. Cancer, 79, 1717-1722.

Dolan, P. (1997). Modeling valuations for EuroQol health states. Med Care,,35, 1095-108.

Ebbeling, C.B., Ward, A., Puleo, E.M., Widrick, J. & Rippe, J.M. (1991) Development of a single-stage submaximal treadmill walking test. Med Sci Sports Exerc, 23, 966-973.

EuroQoL: Group EuroQoL – a new facility for the measurement of health-related quality of life. Health Policy 1990, 16:199-208.

Fong, D. Y., Ho, J. W. C., Hui, B. P. H., Lee, A. M., Macfarlane, D. J., Leung, S. S. K. et al. (2012). Physical actvity for cancer survivors: meta-analysis of randomised-controlled trials. BMJ, 344.

Galvao, D.A. & Newton, R.U. (2005) Review of exercise intervention studies in cancer patients. J Clin Oncol, 23, 899-909.

Godin, G.& Shephard, R. J. (1997) Godin Leisure-Time Exercise Questionnaire. Medicine and Science in Sports and Exercise. 29 June Supplement: S36-S38.

Groeneveldt, L. et al. "A Mixed Exercise Training Programme Is Feasible and Safe and May Improve Quality of Life and Muscle Strength in Multiple Myeloma Survivors." BMC Cancer, 13, 31. doi:10.1186/1471-2407-13-31.

Guiliani, N., Rizzoli, V., & Roodman, G. D. (2006). Multiple myeloma bone disease: pathophysiology of osteoblast inhibition. Blood, 108, 3992-3996.

Joffe, J., Williams, M.P., Cherryman, G.R., Gore, M., McElwain, T.J. & Selby, P. (1988) Magnetic resonance imaging in myeloma. Lancet, 1, 1162-1163.

Kyle, R.A, Rajkumar SV. (2008) Multiple myeloma. Blood. 111, 2962-2972.

Jones, L.W., Courneya, K.S., Vallance, J.K., Ladha, A.B., Mant, M.J., Belch, A.R., Stewart, D.A. & Reiman, T. (2004) Association between exercise and quality of life in multiple myeloma cancer survivors. Support Care Cancer.

Kraemer, W. J. & Ratamess, N. A. (2004). Fundamentals of Resistance Training: Progression and Exercise Prescription. Medicine and Science in Sports and Exercise, 36, 674-688.

Kumar, S.K. et al. (2008). Improved survival in multiple myeloma and the impact of novel therapies. Blood, 2008 111, 2516-2520.

Kumar Shaji K, S Vincent Rajkumar, Angela Dispenzieri, Martha Q Lacy, Suzanne R Hayman, Francis K Buadi, Steven R Zeldenrust, et al. "Improved Survival in Multiple Myeloma and the Impact of Novel Therapies." Blood 111, no. 5 (March 1, 2008): 2516–2520. doi:10.1182/blood-2007-10-116129.

Kvam, K., Fayers, P.F. & Wisloff, F. (2011). Responsiveness and minimal important score differences in quality-of-life questionnaires: a comparison of the EORTC QLQ-C30 cancer-specific questionnaire to the generic utility questionnaires EQ-5D and 15D in patients with multiple myeloma. European Journal of Haematology, 87, 330-337.

Kyle, R. A. & Rajkumar, S. V. (2008). Multiple myeloma. Blood, 111, 2962-2972.

Lorig K, Stewart A, Ritter P, González V, Laurent D, & Lynch J, Outcome Measures for Health Education and other Health Care Interventions. Thousand Oaks CA: Sage Publications, 1996, pp.24-25,41-45.

Lucia, A., Earnest, C., & Perez, M. (2003). Cancer-related fatigue: can exercise physiology assist oncologists? Lancet Oncology, 4, 616-625.

MacDougall, J.D., Tarnopolsky, M.A., Chesley, A. & Atkinson, S.A. (1992) Changes in muscle protein synthesis following heavy resistance exercise in humans: a pilot study. Acta Physiol Scand, 146, 403-404.

Mirels, H. (1989) Metastatic disease in long bones. A proposed scoring system for diagnosing impending pathologic fractures. Clin Orthop, 256-264.

Mishra, S. I., Scherer, R. W., Geigle, P. M., Berlanstein, D. R., Topalogu, O., Gotay, C. C. et al. (2012). Exercise interventions on health-related quality of life for cancer survivors (Review). Cochrane Reviews, 1-361.

Nieman, D.C. (2003) Current perspective on exercise immunology. Curr Sports Med Rep, 2, 239-242.

Oken, M. M. (1982). Toxicity and response criteria of the Eastern Cooperative Oncology Group. Am J Clin Oncol, 5, 649-55.

Royston, P. & White, I. (2011). Multiple imputation by chained equations (MICE): implementation in STATA. J Stat Software, 45 (4), 1-20.

Sadeh, A., Sharkey, K. M., & Carskadon, M. A. (1994). Sleep, 17, 201-207.

Schmitz, K. H., Courney, K. S., Matthews, C., Denmark-Wahnefried, W., Galvao, D. A., Pinto, B. M. et al. (2010). American College of Sports Medicine Roundtable on Exercise Guidelines for Cancer Survivors. Medicine & Science in Sport and Exercise, 42, 1409-1426.

Schneider, J.K., Eveker, A., Bronder, D.R., Meiner, S.E. & Binder, E.F. (2003) Exercise training program for older adults. Incentives and disincentives for participation. J Gerontol Nurs, 29, 21-31.

Shroder, F.H. et al. (2009) Screening and prostate cancer mortality in a randomized European study. NEJM, 360, 1320-28

Smith, MR, Eastham J, Gleason, J., Shasha D., Simon, T., Zinner, N. (2003) Randomized controlled trial of zoledronic acid to prevent bone loss in men receiving androgen deprivation for nonmetastatic prostate cancer. J Urol, 169, 2008-2012.

Steins Bisschop CN, Velthuis MJ, Wittink H, Kuiper K, Takken T,. van der MeulenW JTM, Lindeman E, Peeters P HM and May AM. Cardiopulmonary exercise testing in cancer rehabilitation: a systematic review. Sports Medicine 42: 367-379, 2012.

Strickland, G. Pauling, J. Cavill, C. McHugh, N. (2012). Predictors of health-related quality of life and fatigue in systemic sclerosis: evaluation of the EuroQol-5D and FACIT-F assessment tools. Clin Rheumatol, 31, 1215-1222.

van Remoortel, H., Raste, Y., Louvaris, Z., Giavedoni, S., Burtin, C., Langer, D. et al. (2012). Validity of six activity monitors in chronic obstructive pulmonary disease: A comparison with indirect calorimetry. PLoS ONE, 7.

Terpos, E. et al. (2004). Autologous stem cell transplantation normalizes abnormal bone remodeling and sRANKL/osteoprotegerin ratio in patients with multiple myeloma. Leukemia, 18, 1420–1426.

Terpos, E. et al. "The use of biochemical markers of bone remodeling in multiple myeloma: a report of the International Myeloma Working Group". Leukemia 24, 1700-1712 (October 2010).

Webster, K., Cella, D., & Yost, K. (2003). The Functional Assessment of Chronic Illness Therapy (FACIT) Measurement System: properties, applications and interpretation. Health and Quality of Life Outcomes, 1, 79.

Woo, E., Yu, Y.L., Ng, M., Huang, C.Y. & Todd, D. (1986) Spinal cord compression in multiple myeloma: who gets it? Aust N Z J Med, 16, 671-675.

Zigmond A. S. & Snaith, R. P. (1983). The Hospital Anxiety and Depression Scale. Acta Psychiatrica Scandinavica, 67, 361-370

Protocol appendix 1. Modified International Myeloma Working Group Uniform Criteria of Response and Progression

(Blade et al, 1998 & Durie et al, 2006; Rajkumar et al, 2011)

Paraprotein responses should only be calculated using sequential paraprotein measurements made in the same laboratory using the same method

All response categories require 2 consecutive assessments made at any time before the institution of any new therapy. All categories also require no known evidence of progressive or new bone lesions if radiographic studies were performed. Radiographic studies are not required to satisfy these response requirements

Complete Response (CR) requires all of the following:

1. Absence of the original monoclonal paraprotein in serum / urine by routine electrophoresis and immunofixation. The presence of oligoclonal bands consistent with oligoclonal immune reconstitution does not exclude CR.
2. <5% plasma cells in a bone marrow (confirmation with repeat bone marrow is not needed).
3. No increase in size or number of lytic bone lesions on radiological investigations, if performed (development of a compression fracture does not exclude response).
4. Disappearance of soft tissue plasmacytomas.
5. For patients with light chain myeloma (the serum and urine M-protein are unmeasurable), a normal FLC ratio of 0.26 to 1.65 (or laboratory-specific normal FLC ratio reference range) in addition to the CR criteria above.

Patients in whom some, but not all of the criteria for CR are fulfilled are classified as VGPR. This includes patients in whom electrophoresis is negative but in whom immunofixation has not been performed.

Very Good Partial Response (VGPR)

1. Serum and urine M-protein detectable by immunofixation but not on electrophoresis

or

2. ≥90% reduction in serum M protein level plus urine light chain excretion <100mg per 24h.
3. No increase in size or number of lytic bone lesions on radiological investigations, if performed
4. For patients with light chain myeloma (the serum and urine M-protein are unmeasurable), >90% decrease in the difference between involved and uninvolved FLC levels

Partial Response (PR)

1. ≥50% reduction in the level of the serum monoclonal paraprotein level, and
2. Reduction in 24-hour urinary light chain excretion either by a ≥90% or to <200 mg per 24h, if measured
3. For patients with light chain myeloma (serum and urine M protein are unmeasurable), ≥50% reduction in the difference between involved and uninvolved serum FLC levels
4. For patients with non-secretory myeloma only, ≥50% reduction in plasma cells, in a bone marrow, provided baseline percentage was ≥30%
5. In addition, ≥50% reduction in the size of soft tissue plasmacytomas, if present at baseline
6. No increase in size or number of lytic bone lesions on radiological investigations, if performed.

Patients in whom some, but not all of the criteria for PR are fulfilled are classified as MR.

Minimal Response (MR) requires all the following

1. 25-49% reduction in the level of the serum monoclonal paraprotein level
2. 50-89% reduction in 24-hour urinary light chain excretion, which still exceeds 200 mg/24h
3. For patients with non-secretory myeloma only, 25-49% reduction in plasma cells in bone marrow
4. 25-49% reduction in the size of soft tissue plasmacytomas by radiological investigations if performed
5. No increase in size number of lytic bone lesions on radiological investigations, if performed.
6. MR also includes patients in whom some, but not all, the criteria for PR are fulfilled,

No change (NC)

Not meeting the criteria of either minimal response or progressive disease

Progressive Disease (PD) requires one or more of the following:

1. ≥25% increase from lowest response in serum monoclonal paraprotein level which must also be an absolute increase of at least 5g/L and confirmed by at least one repeated investigation.
2. ≥25% increase from lowest response level in 24-hour urinary light chain excretion, if measured, which must also be an absolute increase of at least 200mg/24h and confirmed by at least one repeated investigation.
3. For patients with light chain myeloma (the serum and urine M-protein are unmeasurable), ≥ 25% increase from the lowest response level in the difference between involved and uninvolved serum FLC levels. The absolute increase must be > 100 mg/L

4. ≥25% plasma cells in bone marrow, which must also be an absolute increase of at least 10%
5. Definite increase in the size of existing bone lesions or soft tissue plasmacytomas.
6. Development of new lytic bone lesions or soft tissue plasmacytomas. Development of a compression fracture does not exclude response.
7. Development of hypercalcemia, corrected serum calcium >11.5mg/dL or 2.8 mmol/L, not attributable to any other cause

Relapse from CR requires at least one of the following:

1. Reappearance of serum or urinary paraprotein on routine electrophoresis or on immunofixation confirmed by at least one further investigation and excluding oligoclonal immune reconstitution
2. ≥5% plasma cells in bone marrow
3. Development of new lytic bone lesions or soft tissue plasmacytomas or definite increase in the size of residual bone lesions. Development of a compression fracture does not exclude continued response.
4. Development of hypercalcaemia (corrected >2.8mmol/L) not attributable to any other cause.

Protocol appendix 2. ECOG Criteria

**ECOG PERFORMANCE STATUS***	
**Grade**	**ECOG**
0	Fully active, able to carry on all pre-disease performance without restriction
1	Restricted in physically strenuous activity but ambulatory and able to carry out work of a light or sedentary nature, e.g., light house work, office work
2	Ambulatory and capable of all selfcare but unable to carry out any work activities. Up and about more than 50% of waking hours
3	Capable of only limited selfcare, confined to bed or chair more than 50% of waking hours
4	Completely disabled. Cannot carry on any selfcare. Totally confined to bed or chair
5	Dead

* As published in Oken et al (1982).

Protocol appendix 3. Eligibility & radiological screening of patients

Eligible patients will undergo a general physical examination, resting ECG where clinically indicated, laboratory investigations and radiological evaluation. Those with spinal instability and high risk of fracture (see below) or cardiovascular disease that precludes exercise will be excluded.

Patients will have a full skeletal survey, which should include a postero-anterior (PA) view of the chest, antero-posterior (AP) and lateral views of the cervical spine (including an open mouth view), thoracic spine, lumbar spine, humeri and femora, AP and lateral views of the skull and AP view of the pelvis. In addition, any symptomatic areas should be specifically visualized with appropriate views. A scoring system based on clinical and radiological findings (Table 1) has been devised to predict the likelihood of fracture in long bones affected by metastatic disease (Mirels 1989). In this study, patients who score 8 or more will be excluded.

Table 1. Scoring system for diagnosing impending pathological fractures.

	Score		
Variable	1	2	3
Site	Upper limb	Lower limb	Peritrochanteric
Pain	Mild	Moderate	Functional
Lesion	Blastic	Mixed	Lytic
Size	<1/3	1/3-2/3	>2/3

A score of 7 or less (5% probability of fracture) suggests a low probability of fracture, such that conservative management (chemo/radiotherapy) is appropriate.

A score of 8 (15% probability of fracture) is slightly suggestive of impending fracture. In this situation, the relative benefits of surgery need to be weighed against the risk of fracture in the individual patient.

A score of 9 or more (33% probability of fracture) is diagnostic of impending fracture, and indicative of prophylactic fixation of the bone.

Up to 70% of patients with myeloma experience vertebral collapse during the course of their disease (Carson*, et al* 1955), with involvement most frequent in the lower thoracic and lumbar vertebrae. Spinal cord compression secondary to vertebral body collapse occurs in up to 25% patients with vertebral body collapse (Woo*, et al* 1986). It is common practice to perform a plain X-ray to confirm a suspected vertebral fracture. In the event of suspected cord compression, MR imaging is the technique of choice (Joffe*, et al*

1988). It provides an accurate assessment of the level and extent of cord or nerve root compression, the size of the tumour mass and the degree to which it has extended into the epidural space. In the event of suspected cord compression or back pain that cannot be accounted for by radiological findings on the skeletal survey, an urgent MR scan of the thoracic and lumbar spine will be performed prior to inclusion in study.

Protocol appendix 4. Details of clinical assessments

Haematology:

Full blood count, including white blood cell differential and haematocrit

Biochemistry:

Renal function, liver function, immunoglobulin levels, vitamin D, serum protein electrophoresis and paraprotein quantification

Urinary protein excretion (spot urine) and Bence Jones protein quantification for patients with Bence Jones Myeloma only

Cytokine levels:

Circulating levels of IL-6, TNF and CRP will be measured by ELISA on stored serum samples *

* These tests will be performed in the Haematology Research Laboratory at UCLH, all other tests are part of routine follow up care for these patients

Bone metabolism tests:

These will be performed in the Myeloma Laboratory at UCL Cancer Institute.

Serum levels of OPG and soluble RANKL will be assessed by ELISA (Biomedica, Medizinprodukte, Gesellschaft GmbH & Co, KG, Wien, Austria).

Serum TRACP-5b will be measured using a solid phase immunofixed-enzyme activity assay (Bone TRAP assay, SBA, Oulu, Finland).

Serum basic ALP will be determined by ELISA (Metra BAP, Quidel Corporation, San Diego, CA).

Osteoclacin levels in serum will be measured by ELISA (N/MID Osteocalcin, osteometer Biotech A/S. Herley, Denmark respectively).

Urinary NTX excretion will be measured by ELISA (OSTEOMARK NTX urine, Ostex International, Inc., Seattle, USA).

Protocol appendix 5. Exercise program

Each patient will have a programme drawn up based the findings of the therapist on initial assessment , and individualised based on the patient's current strength and function, personal goals, sites of bone disease and other musculoskeletal problems.

Aerobic Exercise Training

Aerobic training will be in the form of walking; however patients may also exercise on a cycle ergometer, cross-traininer or stepper if it is more appropriate and convenient for them. The aerobic exercise training will start with 10 or 15 min bouts at an intensity appropriate to the patient (eg 50% of heart rate reserve-75% of maximum (HRR) (Courneya, et al 2003). To support monitoring of correct exercise intensity at home, patients will be asked to report their rating of perceived exertion (RPE) using the Borg Scale (See Borg Scale document). Gradual progression in the exercise training will be achieved by alternately increasing exercise duration by 5 min and exercise intensity by 5% heart rate reserve (HRR) every 4 weeks, or as appropriate. This will result, eg in an exercise session of 30 min duration at an intensity of 60-75% HRR of maximum in the final 4 weeks of the programme.

An example of an exercise programme is as follows:

1. Stretches and warm up

- Gentle group work with gentle warm up and 3-4 LL and UL stretches

2. Lower limb exercises

- Leg press 3 sets 10RM – single leg (1 warm up set)
- Squats 3 sets to fatigue – single leg
- calf raises on step single leg – 3 sets to fatigue
- step ups on bench – 3 sets to fatigue on each leg

3. Upper limb strengthening

- push ups – wall / kneeling/ full
- shoulder press – hand weights – 3 sets to fatigue
- upright Row – hand weights – 3 sets to fatigue
- pull downs

4. Trunk strengthening (pilates type exercises)

- Transversus contraction + pelvic tilts + leg lifts
- Transversus mat work with Unilat arm and leg exercise → bridging 2L→ 1 L
- theraband sling work R & L leg – progr R & L arm –progr rotation
- kneeling arm and leg lifts → elbows knees → elbows / toes → plank (front/side/ rotation)

2-4 will be performed as a self timed ex or as a timed circuit

5. Aerobic fitness (work levels outlined in protocol)

- walking on treadmill
- Step machine
- Cross Trainer
- Cycle ergometer

Patients will be given an exercise sheet (See below) to complete for each session (home and supervised). This will be used to monitor the exercise times/ loads / weights they use for both home and supervised sessions, and will include a borg scale rating for each exercise.

(Borg and Borg 2002; Borg and Kaijser 2006; Borg and DAHLSTROM 1962; Borg 1982)

Progression will be achieved by increasing the resistance or the number of repetitions performed on each exercise. Again, all exercise programmes will be prescribed on an individual basis for each patient to ensure suitability and to promote adherence to the programme.

Safety

To minimise the risk of fracture and increasing bone pain in this patient group, all patients will undergo radiological screening as detailed under Appendix 3. Patients will be given an induction to the class where they will be shown the exercises and the study physiotherapist will correct their technique and be present to answer questions. Patients will be warned about pain and swelling with exercise, and will also be told to consult the physiotherapist prior to exercising if they feel pain or other untoward symptoms such as diziness. Due to bone risks with exercise, all exercises will be commenced at a level that the patient can manage without pain, and progressions will be gradual . If a patient has a febrile illness or fatigue levels are too great – they will be asked to skip the session for that time point.

Water is provided in the gym and patients will be asked to ensure they have had breakfast (or lunch) on the days of the class and questioned re hydration , and encouraged to drink after exercise. Physiotherapists will follow the pre existing hospital health and safety proceedures of the UCLH and physiotherapy gym. Physiotherapists will have CPR and manual handling training in line with trust policy and evacuation / emergency assistance is available from the other physiotherapy staff who will be present in the dept during the class times.
